# Deciphering the mechanistic roles of ADARs in cancer pathogenesis, tumor immune evasion, and drug resistance

**DOI:** 10.3389/fimmu.2025.1621585

**Published:** 2025-08-07

**Authors:** Xiaoke Wang, Fei Yin, Yangfang He, Yue Qiao, Yan Yan, Jingru Ma

**Affiliations:** ^1^ Department of Neurosurgery, The Second Hospital of Jilin University, Changchun, China; ^2^ Department of Neurology, The Second Hospital of Jilin University, Changchun, China; ^3^ Department of Endocrinology and Metabolism, The Second Hospital of Jilin University, Changchun, China; ^4^ Department of Physical Examination Center, The Second Hospital of Jilin University, Changchun, China; ^5^ Department of Endocrinology, The Second Hospital of Jilin University, Changchun, China; ^6^ Department of Laboratory Medicine, The Second Hospital of Jilin University, Changchun, China

**Keywords:** RNA editing, ADARs, cancer pathogenesis, immune evasion, immunotherapy

## Abstract

RNA is a fundamental biological macromolecule that undergoes several post-transcriptional modifications, including adenosine to inosine (A-to-I) editing by adenosine deaminases acting on RNA (ADARs). These essential enzymes catalyze the conversion of A-to-I in double-stranded RNA (dsRNA) molecules, influencing RNA stability, splicing, and translation, all of which impact various cellular functions. More recently, RNA editing has emerged as a pivotal mechanism in cancer biology, where ADARs, primarily ADAR1 and ADAR2, exert context-dependent roles as either oncogenic drivers or tumor suppressors. Beyond their catalytic editing function, ADARs also regulate cancer-relevant pathways through editing-independent mechanisms, including RNA binding and protein-protein interactions. Dysregulated ADAR activity facilitates carcinogenesis by altering oncogene expression, impairing tumor suppressor pathways, and reprogramming the transcriptome to promote tumor progression. Furthermore, RNA editing may contribute to tumor cell immune evasion by affecting interferon signaling and altering neoantigen presentation, as well as modulating immune surveillance. Additionally, ADAR-mediated RNA modifications contribute to therapy resistance by modifying drug targets and pathways involved in cell survival and repair. This review comprehensively analyzes the multifaceted roles of RNA-editing ADAR enzymes in cancer pathogenesis, emphasizing editing-dependent and -independent mechanisms contributing to tumor progression, immune evasion, and resistance to therapy. Moreover, we highlight the potential of ADARs as prognostic biomarkers and promising therapeutic targets in oncology. This review aims to spark novel precision oncology and cancer immunotherapy strategies by bridging molecular insights with translational applications.

## 1 Introduction

Cancer remains one of the most formidable challenges in modern medicine, as its complexity and heterogeneity pose significant obstacles to effective treatment (1, 2). Cancer is caused by genetic and epigenetic changes that enable cells to multiply and bypass systems that typically govern their survival and migration. Several of these alterations correspond to signaling pathways that regulate cell growth and division, cell death, cell fate, and cell motility. They can be contextualized within the broader framework of disrupted signaling networks that contribute to cancer progression, including tumor microenvironment (TME) alterations, angiogenesis, and inflammatory processes (3, 4). Despite significant advancements in immunotherapy, which has greatly improved cancer treatment by utilizing the immune system to target malignant cells, resistance to these therapies remains a critical challenge (5, 6). A significant body of documentation indicates that tumor immune evasion mechanisms significantly restrict the effectiveness of immunotherapeutic strategies. Tumors can evade immune system attacks through several mechanisms, including the restriction of antigen recognition, the inhibition of immune responses, and the induction of T cell exhaustion (7). Among the emerging molecular mechanisms contributing to cancer progression, RNA editing has gained significant attention as a critical post-transcriptional regulatory process (8–12). RNA editing, particularly A-to-I editing mediated by the ADAR1 enzyme, plays a pivotal role in shaping the transcriptomic landscape of cancer (8–10, 12). ADAR1-mediated RNA editing influences diverse biological processes, including RNA stability (13) and splicing (14, 15), while also modulating immune responses (16–18). Recent studies have unveiled both RNA editing-dependent and non-catalytic roles of ADAR1 in cancer pathogenesis, including its activity as an RNA-binding protein and its participation in protein-protein interactions, which can modulate oncogenic signaling even in the absence of deaminase function (19, 20).

Two ADARs, namely ADAR1 and ADAR2, are catalytically active (21), while ADAR3, believed to be catalytically inactive (22), is encoded by the genomes of mammals. A-to-I RNA editing is a post-transcriptional mechanism that preferentially converts A-to-I in dsRNA substrates (23). ADAR1 is extensively researched for its crucial role in immune regulation and cancer development (9, 17, 24), while ADAR2 also functions in RNA editing events that influence carcinogenesis and treatment resistance (25, 26). These enzymes significantly influence the transcriptome profile of cancer cells and their interactions with the immune system. Under typical physiological conditions, ADARs are crucial for preserving cellular homeostasis by inhibiting the inappropriate activation of innate immune sensors, including MDA5 (melanoma differentiation-associated protein 5) and protein kinase R (PKR), which identify unedited dsRNAs as foreign entities and initiate antiviral immune responses (27, 28). Tumor development, immune escape, and treatment resistance are all significantly impacted by the widespread RNA editing events caused by the dysregulation of ADAR activity in cancer (29–31). The immune-editing capacity facilitates tumor survival and contributes to the development of resistance to immunotherapies, such as immune checkpoint inhibitors (32). Although ADAR2 has not been studied to the same extent, it is linked to cancer advancement because it edits significant transcripts involved in cancer etiology (25, 33). This review aims to explore comprehensively the mechanistic roles of ADARs in cancer pathogenesis, tumor immune evasion, and drug resistance. By synthesizing recent findings from preclinical and clinical studies, we will elucidate how ADAR-mediated RNA editing contributes to cancer progression and shapes the immune landscape of tumors. We will also discuss the potential of targeting ADARs to overcome immunotherapy resistance and improve patient outcomes, providing new insights into the evolving field of molecular cancer immunology.

### 1.1 Structure, expression, and function of ADAR

The ADAR gene family is extensively conserved throughout metazoans. In vertebrates, three members of the ADAR gene family have been identified: ADAR1, ADAR2, and ADAR3 (34). ADARs comprise a family of enzymes essential for post-transcriptional RNA editing, specifically the conversion of A-to-I in dsRNA (35). The A-to-I conversion may impact numerous biological activities, including recoding RNA, establishing or removing RNA splicing sites, altering RNA structure, and impairing dsRNA pairing (36). Mammals express three ADAR isoforms—ADAR1, ADAR2, and ADAR3—each characterized by a conserved deaminase domain at the C terminus and multiple double-stranded RNA-binding domains (dsRBDs) at the N terminus (21).

**Figure 1:**
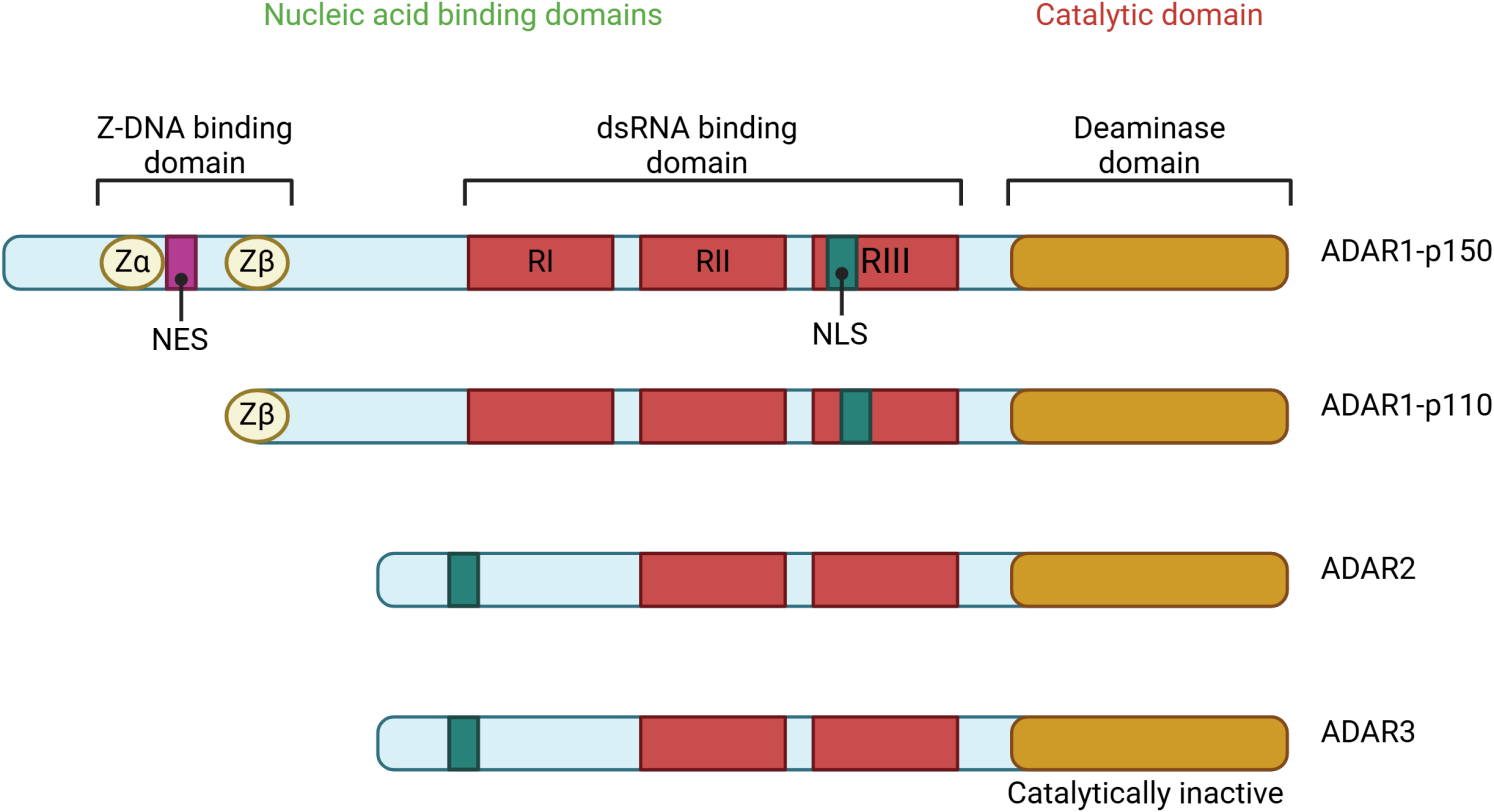
The ADAR protein family consists of several members, including ADAR1, which exists in two forms: the interferon-inducible p150 isoform and the constitutively expressed p110 isoform, as well as ADAR2 and ADAR3. Among these, ADAR1 and ADAR2 exhibit deaminase activity, which is crucial for their role in RNA editing. In contrast, ADAR3 lacks deaminase activity and primarily regulates RNA editing processes. The structural domains of these proteins include Z-DNA binding domains (Zα and Zβ), represented as yellow circles; dsRNA binding domains (RI, RII, and RIII), shown as blue rectangles; and the deaminase catalytic domains, depicted as purple ovals. Notably, a striped purple oval indicates the absence of deaminase activity, as seen in ADAR3. This illustration highlights the domain architecture and functional distinctions within the ADAR protein family.

Furthermore, ADAR1 possesses Z-DNA-binding domains (Z-α and Z-β) in its N-terminal region (37). ADAR1 and ADAR2 possess well-defined adenosine deaminase activity, but ADAR3 lacks deaminase activity and has undefined roles (23). An active-site zinc ion is present in ADARs, coordinated by two cysteines and one histidine from the enzyme. The fourth ligand is a water molecule that attacks the carbon-6 of the adenine base, ultimately releasing the 6-amino group and producing inosine (**Figure 1**) (38). In higher eukaryotic organisms, two ADARs are catalytically active. These ADARs, ADAR1 and ADAR2, have modular domain structures that are similar to each other. Since deleting either of these ADARs in mice results in death, both are essential for life (39).

One of the most extensively researched RNA editing enzymes, ADAR1, is involved in various biological processes (40). The Adar1 gene in humans is located on chromosome 1q21.3. This gene encodes two distinct isoforms, each using different promoters and start codons. The shorter Adar1 isoform encodes a 110 kDa protein (p110) that is consistently and universally produced. The extended isoform encodes a protein of 150 kDa (p150) that is stimulated by interferon (IFN) and features two N-terminal Z-DNA-binding domains, which are not present in p110. Both p110 and p150 can translocate between the nucleus and cytoplasm; however, p110 predominantly resides in the nucleus, while p150 is primarily located in the cytoplasm (41). The p150 isoform suppresses innate immune responses to dsRNA, which can be misrecognized and activate immune cells (42). The p110 isoform may also play a role in suppressing dsRNA sensing and could be involved in cancer cell survival and resistance to the immune response (42, 43).

ADAR1 is widely present in nature and performs A-to-I RNA editing at millions of sites throughout the human transcriptome (44). The expression of ADAR1 is notably high in both fetal and adult hearts, as well as in blood vessels. ADAR1 is necessary for maintaining cardiac homeostasis and function in the adult heart (45). In contrast to ADAR1, ADAR2 expression is limited, with peak levels observed in the brain and central nervous system (46). ADAR2 is often expressed lower than ADAR1 in peripheral organs, such as the heart (46). ADAR2 is believed to operate predominantly in the brain, with its clinical relevance linked to neurological functions, including the editing of aminomethylphosphonic acid receptors, disturbances of which may result in persistent seizures and mortality (47).

The primary biological activity of ADAR1 involves RNA editing, namely the conversion of A-to-I in dsRNA substrates at the post-transcriptional stage. Ribosomes and RNA polymerase recognize inosine as guanosine (G), which replaces RNA base A with G during the transcription process (48). Isosine can form a base pair with cytidine. Inosine, which is chemically similar to guanosine but lacks its exocyclic amine, is often recognized by the cell’s machinery as guanosine, resulting in an A-to-G transition at the RNA level. ADARs are responsible for initiating a process known as base flipping, which involves the specific adenosine being extracted from the dsRNA helix and transferred into the ADAR active site (49). Over many years, it has been proven that ADAR1 plays a role in the innate immune system. ADAR1 modulates innate immunity by inhibiting the pattern recognition receptor (PRR) pathway. Additionally, the activation of ADAR1 reduces the production of interferons and antiviral responses that are mediated by IFN (50). The researchers Zhang et al. found that ADAR1 suppresses endogenous Z-RNAs and identified ZBP1-mediated necroptosis as a novel mechanism influencing the immunogenicity of ADAR1-masked tumors (51). The lack of specific small-molecule inhibitors of ADAR1 is a barrier to the clinical translation and invention of medicinal products for malignancies and autoimmune disorders, which will be significantly important in the future.

The human ADAR2 gene is mapped to chromosome 21, band q22.3 (52), using a method based on polymerase chain reaction. This particular area of chromosome 21 has been linked to many hereditary diseases. The ADAR2 protein produces two major isoforms. These are ADAR2S, which is also known as ADAR2a, and ADAR2L, which is also referred to as ADAR2b (53). ADAR1 and ADAR2 isoforms vary significantly in their structural makeup, notably concerning the number of dsRNA-binding domains and the amino-terminal extension of the ADAR enzyme. This difference is particularly noticeable in the former case. There are only two dsRNA-binding motif repetitions in the N-terminal region of ADAR2, and it lacks a Z domain. Furthermore, it is not affected by IFN stimulation (53). ADAR2 exhibits greater conservation than ADAR1, with its homologous sequence discernible in the Drosophila genome (54). ADAR2 has two copies of the RNA-binding domain in the N-terminal region. On the other hand, the deaminase catalytic domain is located near the C-terminal region. The ADAR2 protein primarily mediates site-specific A-to-I editing in mammalian cells (55).

ADAR3 is expressed only in the nervous system, with the hippocampus and amygdala having the highest levels of expression (22, 56). However, there is no discernible deaminase catalytic activity in wild-type (WT) ADAR3 (57, 58). Five amino acid substitutions have been identified in human ADAR3 that may confer enzymatic activity: A389V, V485I, E527Q, Q549R, and Q733D (57). The association of wild-type ADAR3 with dsRNA structures can influence the editing efficacy of ADAR2, as demonstrated by ADAR3’s suppression of GluR-B pre-mRNA editing in glioblastoma (GBM) cells (59).

## 2 ADARs in health and diseases

ADAR enzymes are vital in safeguarding cellular and systemic health through RNA editing, in which the A-to-I mechanism is predominant (60–62). Dysregulation of ADAR activity may lead to a panoply of diseases, including autoimmune diseases (63, 64), neurological diseases (65, 66), cancer (9, 10, 24), cardiovascular diseases (67–69), and infectious diseases (70, 71). Understanding the role of ADAR in health and disease points to its therapeutic potential. In addition, ADAR plays a critical role in neuronal function and development (72, 73), immune homeostasis (74, 75), RNA stability and diversity (76, 77), and control of the stress response (78, 79). ADARs are essential in distinguishing between self and non-self RNA within cells. To avoid recognition as foreign and to prevent improper activation of the innate immune responses, ADARs edit endogenous dsRNA (80, 81). This is crucial for maintaining immune tolerance and preventing autoimmunity.

Beyond their well-established catalytic activity, ADAR enzymes, especially ADAR1, exert several critical editing-independent functions that significantly impact cancer biology. These include interference with innate immune sensors such as PKR and ZBP1 (28, 82, 83) and modulation of microRNA (miRNA) biogenesis by interacting with pri-miRNAs (84, 85). Notably, these non-catalytic roles have been shown to promote tumor immune evasion, support cancer stemness, and contribute to therapy resistance. For example, ADAR1 facilitates tumor progression even when its deaminase activity is inactivated, as demonstrated by its role in suppressing interferon signaling and modulating necroptosis pathways through Z-RNA binding and interaction with ZBP1 (51). These findings highlight the importance of considering ADARs as RNA editors and multifunctional regulators in the oncogenic landscape.

Chen et al. demonstrated that ADAR1 plays a critical role in human embryonic stem cell (hESC) differentiation and neural induction by regulating miRNA processing independently of its RNA-editing activity (73). ADAR1 deficiency disrupts miRNA and mRNA expression, including the upregulation of self-renewal-related miRNAs like miR-302s, without a significant contribution from its editing function. Genome-wide analyses reveal that ADAR1 binds directly to pri-miRNAs, interfering with their processing as an RNA-binding protein (73). Restoration of normal miRNA expression and differentiation phenotypes was achieved using an enzymatically inactive ADAR1 mutant, underscoring its non-catalytic regulatory role (73). In the case of rheumatoid arthritis (RA), for example, Vlachogiannis et al. conducted research that examined the functions of A-to-I RNA editing mediated by ADAR enzymes (64). ADAR1, particularly its p150 isoform, was shown to be significantly overexpressed in synovial tissues and blood samples collected from patients with RA. This finding aligns with the fact that increased A-to-I editing of certain genes, such as cathepsin S and TNF receptor-associated factors, has occurred. The observed editing rates and ADAR1p150 expression decreased following adequate treatment, indicating a correlation between inflammatory gene regulation and therapeutic response (64). The findings highlight A-to-I RNA editing as a potential therapeutic target in rheumatoid arthritis. In summary, ADARs are essential for maintaining RNA homeostasis and play significant roles in both health and disease. Comprehending the mechanisms that govern their function and dysregulation paves the way for advancing novel treatments for various conditions.

### 2.1 The mechanistic role of ADARs in cancer

**Figure 2:**
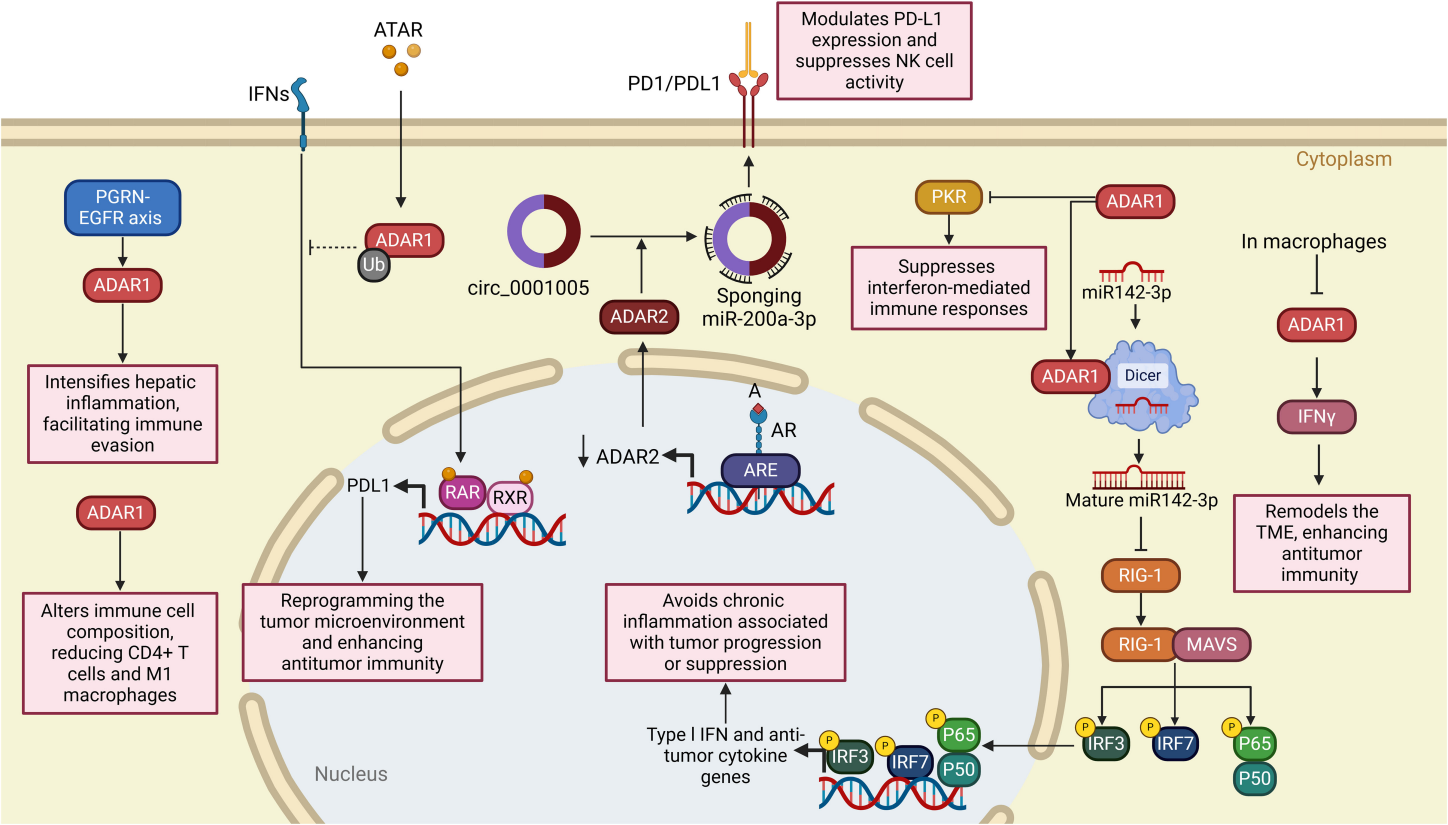
Dual roles of ADARs in cancer progression across multiple tumor types. The schematic illustrates the context-dependent functions of ADAR1 and ADAR2 enzymes in various malignancies, highlighting both pro-tumorigenic (left panel) and tumor-suppressive (right panel) mechanisms. In CRC, HCC, GBM, BC, and AML, ADAR1 promotes oncogenesis via pathways such as FAK/AKT signaling, angiogenesis, immune evasion, and WNT/β-catenin activation, often through editing-dependent and -independent mechanisms. In contrast, in GBM and GC, ADAR2 exerts tumor-suppressive functions through miRNA regulation, inhibition of proliferation pathways (e.g., CDC14B/Skp2/p21 axis), and repression of metastasis-related genes (e.g., PODXL). The interplay between ADAR1 and METTL3 further emphasizes editing-independent roles in driving tumor progression or suppression. These findings underscore RNA editing enzymes’ complex and tissue-specific roles in cancer biology.

In cancer, dysregulation of ADAR activity contributes to tumorigenesis, immune evasion, and therapeutic resistance by editing key transcripts involved in critical biological processes. ADARs, particularly ADAR1 and ADAR2, catalyze A-to-I RNA editing, a post-transcriptional modification that alters RNA sequences, structures, and functions. This editing process influences gene expression, protein diversity, and cellular signaling pathways, playing a central role in cancer biology. By editing transcripts involved in cell proliferation, apoptosis, immune responses, and drug metabolism, ADARs enable cancer cells to acquire hallmarks of malignancy, evade immune surveillance, and resist therapeutic interventions. Below, we explore the mechanistic roles of ADARs in cancer, focusing on their contributions to tumor development, progression, and immune modulation while highlighting their context-dependent functions across different cancer types (**Figure 2**, **Table 1**).

**Table 1:** Mechanistic roles and functional impacts of ADARs across cancer types.

ADAR type	Cancer type	Study type	Mechanism	Outcome	Ref.
ADAR1	HCC	Clinical tissue samples and TCGA dataset	Elevated ADAR1 levels are associated with stromal markers such as CLDN5 in the tumor microenvironment (TME).	ADAR1 expression regulates TME interactions, influencing stromal and immune responses in HCC progression and metastasis.	(8)
ADAR1	HCC	Patient specimens, cell culture, and xenograft models	ADAR1 knockdown upregulates Keap1 and downregulates Nrf2, impairing antioxidant responses and increasing ROS accumulation.	ADAR1 suppression reduces tumor proliferation and enhances sensitivity to oxidative stress inducers, providing a potential therapeutic strategy for HCC.	(86)
ADAR2	HCC	HCC samples	Mutations in ADAR2 increase global A-to-I RNA editing levels in oncogenes and TSGs, introducing a mutation-ADAR2-UES-oncogene/TSG-HCC axis.	Increased ADAR2 editing efficiency due to mutations links RNA editing dysregulation with HCC pathogenesis.	(87)
ADAR2	HCC	Clinical samples and Cell lines	Protein recoding by editing COPA (Isoleucine to Valine). Edited COPA inhibits the PI3K/AKT/mTOR pathway via caveolin-1, while unedited COPA promotes tumorigenesis.	Edited COPA acts as a tumor suppressor, while unedited COPA functions as a tumor promoter, indicating dual roles of ADAR2-mediated editing.	(88)
ADAR2	HCC	HCC patients’ tissue and Cell Culture	RNA editing of pri-miR-214 and pri-miR-122, resulting in A-to-I and U-to-C changes. Reduced pri-miR-214 levels decrease miR-214 and increase Rab15 expression.	ADAR2-mediated editing promotes HCC progression by deregulating miRNA biogenesis.	(89)
ADAR1	CRC	TCGA database, cell culture, and xenograft models	ADAR1 inhibits ferroptosis and promotes invasion and migration via FAK/AKT pathway.	Suppressed ferroptosis and enhanced tumor aggressiveness.	(9)
ADAR	CRC	Bioinformatics analysis, tumor tissue samples, Cell lines and cell culture	OGT-mediated glycosylation stabilizes ADAR, enhancing DNA damage repair and chemoresistance.	Increased chemoresistance through improved DNA repair.	(90)
ADAR1	CRC	Cell Culture, xenograft models and bioinformatics analysis	AZIN1 editing promotes tumor angiogenesis via IL-8 upregulation and c-Myc stabilization.	Tumor progression through enhanced angiogenesis.	(91)
ADAR1	Gastric Cancer (GC)	In Silico - Genomic Analysis	R767 mutation disrupts dsRNA interaction and destabilizes ADAR1, impairing its enzymatic function.	Reduced protein stability and immune evasion pathways.	(92)
ADAR2	Gastric Cancer (GC)	Experimental - functional targeted RNA editing Analysis	Acts as a tumor suppressor; suppresses tumorigenicity via specific editing in PODXL gene.	Suppression of tumor growth and progression.	(93)
ADAR1	Breast Cancer (BC)	Cell lines and cell culture	ADAR1 suppresses ferroptosis via miR-335-5p/Sp1/GPX4 pathway.	Increases tumor progression by inhibiting ferroptosis.	(20)
ADAR1	Breast Cancer Brain Metastases (BC-BrM)	Experimental - Humanized Mouse Model and Patient Samples	ADAR1 p150 isoform upregulates WNT/β-catenin pathway and enhances stem cell markers.	Facilitates metastatic niche formation and resistance in brain metastases.	(94)
ADAR1	Breast Cancer (BC)	Cell culture	ADAR1 interacts with KYNU, which is linked to poor prognosis in TNBC.	Promotes aggressiveness and poor prognosis in TNBC.	(95)
ADAR1	Glioblastoma Stem Cells (GSCs)	Primary patient tumors or xenografted tumors cells, *in vivo* tumorigenesis and bioinformatics analysis	ADAR1 RNA editing links ganglioside metabolism to GSC maintenance and tumor progression.	Maintenance of GSCs; potential therapeutic target identified.	(96)
ADAR1	Glioblastoma (GBM)	Human tissues and cell lines and *in vivo* experiments	METTL3-mediated m6A methylation increases ADAR1 protein levels, promoting tumor growth.	Enhanced glioblastoma growth via editing-independent pathways.	(19)
ADAR2	Glioblastoma (GBM)	Human tissues and Cell Culture and ADAR2 Knockdown	ADAR2 protein decrease promotes cell proliferation, migration, and growth in GBM.	Prognostic relevance and association with GBM pathology.	(26)
ADAR2	Glioblastoma (GBM)	Cell lines, human and mouse brain tissues/RNAs	Restores miRNA editing, reducing onco-miRNAs and enhancing tumor-suppressor miRNAs.	Reduction in GBM cell proliferation and migration.	(11)
ADAR2	Glioblastoma (GBM)	Tumor and control tissues and cell line	ADAR2 editing modulates CDC14B/Skp2/p21/p27 axis, suppressing tumor progression.	Inhibition of GBM growth and tumor suppression.	(97)
ADAR3	Lower-Grade Gliomas (LGG)	Patients samples and ADAR3 expression analysis in datasets	ADAR3 expression inversely correlates with glioma grade, acting as a prognostic biomarker.	Better outcomes in LGG patients; potential for stratification and therapy.	(98)
ADAR1	Acute Myeloid Leukemia (AML)	Experimental - Wnt Signaling Pathway and miRNA Biogenesis	ADAR1 modulates Wnt signaling via pri-miR-766 regulation, promoting AML cell survival and proliferation.	Enhances leukemia progression through Wnt signaling.	(99)
ADAR1	TP53-Mutant AML	Cancer cell lines	ADAR1-mediated RNA editing suppresses inflammatory pathways, aiding survival of TP53-mutant AML cells.	Impaired survival of TP53-mutant AML cells.	(100)
ADAR1	Acute Myeloid Leukemia (AML)	Humanized sAML mouse models	Selective inhibition of ADAR1 splicing disrupts LSC maintenance, favoring normal hematopoiesis.	Prolongs survival in AML mouse models.	(101)
ADAR1	Acute Myeloid Leukemia (AML)	Experimental - LSC Niche Remodeling with 3D Bioreactor	Inhibition of ADAR1 remodels LSC-supportive niche, reducing malignant persistence.	Reduces LSC survival and disrupts malignant niches.	(102)
ADAR2	Acute Myeloid Leukemia (AML)	Mice and patients sample	ADAR2 downregulation via RUNX1-ETO fusion impairs RNA editing, promoting leukemogenesis.	Promotes clonogenic growth and leukemia progression.	(103)
ADAR1	T Cell Acute Lymphoblastic Leukemia (T-ALL)	CRISPR-Cas9 - Functional Genomic Screens	Loss of ADAR1 selectively impairs LIC maintenance by deregulating immune-related transcripts.	Potential therapeutic target identified for eliminating LICs.	(43)

#### 2.1.1 Hepatocellular carcinoma

HCC is the primary malignancy of the liver that occurs the most frequently and is also one of the top causes of death globally due to cancer (104, 105). Cirrhosis, hepatitis B or C virus infections, and other etiologies, which include persistent liver inflammation in general, are the most common causes of this condition, often manifesting in the setting of chronic liver disease (106, 107). Despite recent breakthroughs in diagnostic and therapeutic techniques, the prognosis for HCC remains bleak, highlighting the critical need to uncover novel molecular targets and elucidate the underlying processes of HCC progression. In the context of HCC, abnormal RNA editing and dysregulated ADAR expression have been associated with the development of lung cancer (8, 24, 86, 88, 89, 108). For instance, ADAR2 exerts tumor-suppressive effects in HCC primarily by editing dsRNA structures formed by precursor and antisense transcripts of specific microRNAs (89). Liu et al. demonstrated that ADAR2-mediated editing of the antisense transcript complementary to pri-miR-214 leads to reduced levels of mature miR-214, thereby derepressing its direct target, Rab15, a GTPase implicated in vesicular trafficking and endosomal signaling (89). This cascade ultimately promotes tumor growth by enhancing intracellular trafficking of growth factors and receptor recycling, potentiating mitogenic signaling.

Moreover, ADAR2 mediates a critical protein-recoding RNA editing event in the *COPA* gene. Editing at the I164V site, mediated by ADAR2 binding to intronic editing complementary sequences, transforms COPA from a tumor-promoting to a tumor-suppressing isoform (88). Mechanistically, the edited COPA (COPA^I164V) attenuates tumor growth by suppressing the PI3K/AKT/mTOR pathway via downregulation of Caveolin-1 (CAV1), thereby decreasing mTOR-driven cell proliferation and survival (88). In contrast, reduced ADAR2 expression in HCC leads to hypo-editing of COPA, accumulating the unedited, oncogenic COPA isoform that stabilizes CAV1 and fosters a pro-proliferative cellular phenotype (88). This discovery aligns with prior research that has established a connection between RNA editing and cancer (109–111), but it is the first to identify COPA as a protein-coding target that serves two distinct functions. CRISPR/Cas9’s achievements are bolstered by its innovative manipulation of editing sites and its comprehensive mechanistic understanding. Additionally, there is a significant need for further research into COPA’s function in various types of cancer. Careful interpretation is warranted due to potential biases in patient selection and the inherent limitations of cellular and animal models.

Although ADAR2 is often described as a tumor suppressor due to its editing of key regulatory transcripts, such as COPA in HCC, which shifts the function of ADAR2 from oncogenic to tumor suppressive by inhibiting the PI3K/Akt/mTOR pathway (88), its role is not universally suppressive across all cancer types. For instance, Li et al. identified specific mutations in ADAR2 in HCC that increased global A-to-I editing activity, potentially enhancing oncogenic transcript stability and expression (87). This illustrates that the impact of ADAR2 depends heavily on the specific context and editing targets involved. Similarly, recent studies have revealed increased ADAR2 expression and activity in malignant pleural mesothelioma, particularly in BAP1 wild-type tumors (112). This upregulation correlates with enhanced A-to-I RNA editing in 3′-untranslated regions (3′UTRs) and intronic regions, contributing to tumor heterogeneity and progression (112). Functional analyses demonstrated that ADAR2 knockdown in mesothelioma cell lines leads to reduced cell proliferation, altered cell cycle progression, increased sensitivity to antifolate chemotherapy, and upregulation of IFN-I signaling, indicating a multifaceted role in tumor biology and microenvironmental modulation (112).

Conversely, ADAR1 is frequently overexpressed in HCC and promotes tumor growth by sustaining cellular redox balance and maintaining oncogenic homeostasis during oxidative stress (86). It has been demonstrated that oxidative stress plays a significant role as a catalyst in transforming normal cells into malignant phenotypes, primarily through the disruption of genomic integrity (113–116). Wang et al. reported that the knockdown of ADAR1 disrupts the antioxidant defense by modulating the Keap1/Nrf2 axis—a master regulator of cellular redox homeostasis. Specifically, the loss of ADAR1 leads to the upregulation of Keap1, which inhibits Nrf2, resulting in the accumulation of reactive oxygen species (ROS), enhanced apoptosis, and impaired tumor cell proliferation (86). Thus, ADAR1 acts as a pro-survival factor, enabling HCC cells to withstand oxidative microenvironments, a critical feature for tumor expansion in inflammatory and hypoxic liver tissue. Previous investigations that have focused on ADAR1’s function in cancer biology, notably its role in stress responses (79, 117, 118), are consistent with this study’s findings. The reliability of the results is strengthened by utilizing a comprehensive scientific approach, which includes tissue analysis, cell lines, and animal models. However, the sample size of 50 tissue pairs and the reliance on *in vitro* and xenograft models may limit generalizability to broader HCC populations. Research primarily focused on the Keap1/Nrf2 pathway, leaving the potential involvement of other oxidative stress-related mechanisms unexplored. Moreover, the long-term effects of ADAR1 inhibition *in vivo*, including possible side effects, remain uncertain.

ADAR1 expression was observed to be increased in liver hepatocellular carcinoma (LIHC) and downregulated in many renal malignancies, including kidney chromophobe (KICH), kidney renal clear cell carcinoma (KIRC), and kidney renal papillary cell carcinoma (KIRP), with notable prognostic significance. The research underscores the divergent associations between ADAR1 and stromal scores in the tumor microenvironment, exhibiting a positive correlation in KIRC and a negative correlation in LIHC (8). The tight junction protein Claudin-5 (CLDN5) is well recognized for its function in preserving the integrity of endothelial barriers, particularly in vascular endothelial cells and the blood-brain barrier (117). The several tasks that CLDN5 plays in tumor growth, metastasis, and the TME have drawn attention to it in the cancer setting (118–120). Additionally, a distinct correlation was observed between ADAR1 expression and the stromal cell marker CLDN5 in blood endothelial cells (BECs) and lymphatic endothelial cells (LECs), providing a refined perspective on ADAR1’s role across various cancer types (8). The study mentioned earlier supports prior findings that implicate ADAR1 as a key regulator of cancer progression while expanding upon existing knowledge by exploring its interactions with stromal cells and the TME. Although the limited sample size (e.g., 26 KIRC and 30 LIHC samples for IHC) may constrain the generalizability of the results, the innovative integration of stromal and immune microenvironment profiling yields valuable mechanistic insights. While the data reveal a robust correlation between ADAR1 and stromal markers such as CLDN5, establishing a causal relationship remains an open question. Additionally, future work could strengthen these findings by moving beyond associative analyses to investigate ADAR1’s functional mechanisms in TME regulation.

All in all, in HCC, ADARs exhibit context-dependent and sometimes opposing roles in tumor biology. ADAR1 predominantly functions as a proto-oncogene, enhancing tumor cell survival under oxidative stress and contributing to poor prognosis through modulation of the Keap1/Nrf2 axis (86). In contrast, ADAR2 demonstrates a dualistic role: its editing of COPA suppresses tumor progression (88). This complex landscape highlights the non-binary nature of ADAR function in HCC, where RNA editing can either promote or suppress malignancy depending on the cellular and genetic context. A nuanced understanding of these dynamics is crucial for exploiting ADARs as therapeutic targets in HCC.

#### 2.1.2 Colorectal cancer

CRC ranks among the most prevalent types of cancer globally and significantly contributes to the morbidity and mortality associated with this disease (121, 122). The condition originates from the epithelial cells that constitute the lining of the colon and rectum, typically arising due to a progressive accumulation of genetic and epigenetic alterations (123, 124). In recent years, new data have brought to light the dysregulation of the ADAR family of enzymes in CRC, suggesting that these enzymes play a crucial role in tumor growth (9, 125). A higher level of ADAR expression has been linked to worse clinical outcomes in the setting of CRC, suggesting that the enzyme may play a role in the aggressiveness of tumors and the progression of the disease (9, 90, 125). For example, Wei et al. revealed a novel, previously unrecognized angiogenic activity of ADAR1 by editing Antizyme Inhibitor 1 (AZIN1) (91). A-to-I-edited AZIN1 inhibits OAZ2-dependent proteasomal degradation of the oncoprotein c-Myc, thereby leading to the transcription and secretion of the pro-angiogenic cytokine IL-8 (91). This creates a permissive tumor vascular microenvironment that fuels CRC progression. This mechanism positions RNA-edited AZIN1 as a critical angiogenic driver in CRC, highlighting the translational potential of IL-8 antagonists (e.g., reparixin) in hyper-edited tumors (91). Wei et al. determined that RNA-edited AZIN1 plays a significant role in the tumor vascular microenvironment and identifies IL-8 signaling, primarily through the application of small-molecule antagonists, such as reparixin, as a promising therapeutic target in hyper-edited cancer (91). This is consistent with prior research that has linked AZIN1 alterations to more aggressive tumor morphologies in other malignancies (126, 127), but the explicit relationship to angiogenesis through IL-8 has not been thoroughly investigated until now. The importance of c-Myc in the growth of tumors has been well-documented (128, 129), but the focus of this work on the OAZ2-mediated delay of c-Myc degradation provides a fresh perspective on how post-transcriptional changes might influence traditional (91, 130) oncogene pathways. The findings would benefit from validation in larger, more diverse cohorts to ensure broader clinical applicability. Controlling for additional angiogenic mediators beyond IL-8 could help delineate its specific role in tumor vascularization. Future research directions should include a systematic evaluation of IL-8-targeting therapeutics such as reparixin, with particular attention to (1) long-term treatment efficacy, (2) safety profiles across patient subgroups, and (3) emerging resistance patterns in clinical settings.

He et al. demonstrated that ADAR1 is significantly overexpressed in CRC tissues, and its high expression correlates with poor prognosis (9). Mechanistically, ADAR1 enhances tumor proliferation, invasion, and migration by activating the FAK/Akt signaling pathway, a central axis in cytoskeletal reorganization, motility, and cell survival (9). Silencing ADAR1 suppressed tumor growth *in vitro* and *in vivo* and induced ferroptosis, a form of iron-dependent cell death, by attenuating FAK/AKT activation. Notably, both isoforms—ADAR1-p110 and interferon-inducible ADAR1-p150—contributed to this regulation, with ADAR1-p110 playing a predominant role (9).

In summary, ADAR1 emerges as a critical tumorigenic factor in CRC by orchestrating multiple pro-oncogenic mechanisms. A-to-I editing of AZIN1 enhances c-Myc stabilization and IL–8-mediated angiogenesis, establishing a permissive vascular niche for tumor progression. Concurrently, ADAR1 promotes tumor cell survival and metastatic potential via activation of the FAK/AKT axis and suppression of ferroptosis. These dual roles modulating the TME and intracellular survival signaling underscore the multifaceted oncogenic capacity of ADAR1 in CRC. Given its consistent association with poor prognosis, therapeutic targeting of ADAR1 or its downstream effectors represents a promising strategy for managing hyper-edited and treatment-resistant CRC.

#### 2.1.3 Breast cancer

BC continues to pose a considerable challenge, even with progress in early detection and treatment, owing to its individual variability. BC tumors are categorized into four principal intrinsic molecular subgroups, each possessing distinct prognostic and therapeutic implications (131). Triple-negative breast cancer (TNBC) represents the most aggressive variant of BC, often classified as a subtype of basal-like BC (132). Basal-like exhibits a poor prognosis and aggressive tumor biology, presenting limited treatment alternatives (133). Conversely, Luminal A-subtype cancers exhibit the most favorable prognosis and tumor biology when provided with appropriate endocrine therapy (134). Exploring the intricate molecular pathways involved in cancer biology and development may reveal innovative approaches and targets for therapeutic intervention in cancer treatment. Recent findings have elucidated the significant role of ADARs in the pathogenesis of BC (20, 25, 42, 95, 135, 136). Recent work by Binothman et al. has uncovered a novel functional axis involving ADAR1 and kynureninase (KYNU) in TNBC, highlighting a previously underappreciated editing-independent role for ADAR1 in cancer progression (95). Utilizing immunoprecipitation followed by mass spectrometry (IP-MS) in highly aggressive MDA-MB-231 TNBC cells, the study identified KYNU as a direct protein interactor of ADAR1, alongside four other candidates. KYNU is a key enzyme in the kynurenine pathway of tryptophan metabolism, catalyzing the conversion of 3-hydroxykynurenine to 3-hydroxyanthranilic acid—a metabolic route increasingly recognized for its role in immune regulation, redox balance, and tumor immune evasion (95). Notably, KYNU expression was significantly upregulated in TNBC tissues, and its high expression correlated with poor overall survival, supporting its pro-tumorigenic function. The ADAR1–KYNU interaction was functionally relevant, as ADAR1 knockdown reduced KYNU protein levels, suggesting a stabilizing or regulatory role of ADAR1 independent of its RNA editing activity (95). Although the precise mechanism remains to be elucidated, it is plausible that ADAR1 acts as a scaffolding protein, protecting KYNU from proteasomal degradation or facilitating its intracellular localization and function. Moreover, KYNU overexpression in TNBC may drive immune suppression via the production of immunosuppressive kynurenine metabolites (137, 138), which are known ligands of aryl hydrocarbon receptor (AhR) pathways. This metabolic-immunologic crosstalk, supported by the ADAR1–KYNU axis, could enhance tumor cell survival, resistance to immune clearance, and metastatic potential, especially in the immune-cold microenvironment characteristic of TNBC. The ADAR1–KYNU axis represents a promising therapeutic target, warranting further exploration for developing small molecule inhibitors or degradation-inducing strategies to disrupt this interaction in treatment-resistant TNBC.

In another work, Sposito et al. demonstrated that the p150 isoform of ADAR1, induced by interferon, is highly expressed in BC brain metastases (BC-BrM) (136). In patient-derived xenografts and humanized mouse models, ADAR1-p150 activated the WNT/β-catenin signaling pathway, upregulating stemness-associated markers such as CD44 and ALDH1 (136). Knockdown of ADAR1 attenuated these markers, suggesting a crucial role in maintaining the stem-like properties of metastatic cells, essential for colonization of the brain microenvironment and resistance to therapy (136). The findings indicate that targeting ADAR1 may provide treatment options for aggressive and metastatic BC.

Cottrell et al. identified ADAR1-p110’s interaction with DHX9, an RNA helicase that suppresses dsRNA sensing pathways (44). In ADAR1-dependent BC cell lines, the knockdown of DHX9 led to PKR activation and cell death, underscoring a cooperative role between ADAR1 and DHX9 in suppressing innate immune activation and promoting tumor cell survival (139–141). This editing-independent immune modulation facilitates immune evasion and enhances tumor persistence. A small number of BC cell lines were used in the study, which may not represent the complete heterogeneity of BC. Furthermore, the study’s conclusions were derived from *in vitro* studies without any validation in TME. Additionally, the focus on DHX9 overlooks the potential roles of other ADAR1-p110 interactors, limiting the broader implications of the research. Also, the study could examine how these mechanisms operate in different cancer subtypes beyond BC.

Yin et al. uncovered a non-catalytic function of ADAR1 in regulating ferroptosis in BC cells (20). The loss of ADAR1 in MCF-7 and MDA-MB-231 cell lines enhanced intracellular ROS levels, lipid peroxidation, and iron accumulation, indicating increased ferroptosis (142–144). Yin et al. evaluated the impact of knocking down ADAR1 and plasmid-mediated overexpression in MCF-7 and MDA-MB-231 cell lines with CRISPR-Cas9 technology. They also assessed the effects on cell survival, ROS, malondialdehyde (MDA), glutathione (GSH), iron (Fe^2+^), GPX4 protein levels, and miR-335-5p (20). The loss of ADAR1 reduced cell proliferation, GSH, and GPX4 levels while raising ROS, MDA, Fe^2+^, and miR-335-5p. Conversely, ADAR1 overexpression produced the opposite results (20). ADAR1 suppressed ferroptosis by modulating the miR-335-5p/Sp1/GPX4 pathway independently of its deaminase activity, demonstrating an editing-independent regulatory role that influences redox homeostasis and therapeutic resistance (20). These data indicate that ADAR1 promotes BC growth by inhibiting ferroptosis. Previous research has demonstrated ADAR1’s significance in RNA editing and its participation in cancer development via miR-532-5p and METTL3 regulation (145). This work extends previous findings by establishing a connection between ADAR1 and the prevention of ferroptosis. This discovery aligns with the growing interest in ferroptosis as a therapeutic target. It complements existing research by providing a specific mechanistic link to GPX4, a pivotal ferroptosis regulator. The study’s limitations include reliance on only two BC cell lines (MCF-7 and MDA-MB-231), which may not fully represent the heterogeneity of BC. Additionally, the findings are based solely on *in vitro* experiments, lacking *in vivo* validation to confirm the relevance of ADAR1-mediated ferroptosis suppression within the TME. Finally, the study does not explore other potential non-canonical functions of ADAR1 beyond the miR-335-5p/Sp1/GPX4 pathway, leaving gaps in understanding its broader role in cancer biology.

Overall, ADAR1 functions as a multifaceted oncogenic driver in BC, particularly in aggressive subtypes such as TNBC and brain-metastatic disease. Acting beyond its canonical RNA-editing activity, ADAR1 promotes tumor progression through several distinct mechanisms: it sustains cancer stemness and metastatic potential via WNT/β-catenin signaling, inhibits ferroptosis through the miR-335-5p/Sp1/GPX4 axis, and suppresses innate immune activation in cooperation with RNA helicases such as DHX9. Notably, its interaction with the metabolic enzyme KYNU unveils a novel immunometabolic axis that may facilitate immune evasion and support survival within an immune-cold tumor microenvironment. These findings underscore the critical role of ADAR1 in shaping the tumor epigenome, metabolism, and immune landscape, establishing it as a compelling therapeutic target in TNBC.

#### 2.1.4 Gastric cancer

GC is a gastrointestinal malignancy whose incidence has gradually increased. In clinical practice, radical resection is the preferred therapeutic option. When radical resection is combined with a standard chemotherapy treatment regimen, the survival rate for GC patients increases (146); nevertheless, the high mortality rate remains a significant issue for physicians. In recent years, the introduction of targeted therapies has addressed the inadequacies of standard treatment regimens, resulting in ongoing increases in cancer patient survival rates (147). Cancer therapy research increasingly focuses on identifying new targets and understanding their roles in drug resistance mechanisms. Recent studies have indicated that ADARs play a vital role in the etiology of GC (10, 31, 92, 93, 148). For example, one study assessed the role of ADAR-mediated RNA editing in GC, identifying ADAR1 as an oncogene and ADAR2 as a tumor suppressor (93). The abnormal regulation of these enzymes in GC results in a unique RNA misediting phenotype, which correlates with poor patient prognosis. A specific editing event in the PODXL gene demonstrates ADAR2’s functional role in tumor suppression (93). The aforementioned study highlights RNA editing as a critical driver of GC development, suggesting novel treatment targets through ADAR1 suppression or ADAR2 restoration.

Valentine et al. analyzed pan-cancer datasets and discovered that mutations within the dsRNA-binding domain of ADAR1—particularly the R767 substitution impair dsRNA recognition, disrupt immune surveillance, and may promote tumor progression (92). These mutations also exhibit mutual exclusivity with tumor suppressor genes, such as PTEN and BLCAP, suggesting a compensatory oncogenic reliance on ADAR1-mediated pathways when canonical suppressors are lost (92). Epistatic investigations show that ADAR1 mutations are mutually exclusive with genes such as PTEN, Akt1, and BLCAP, which appear to be required for cancer cell survival when ADAR1 is compromised (92). Mechanistically, ADAR1 overexpression promotes immune evasion by editing or masking endogenous dsRNAs, thereby dampening MDA5-dependent interferon signaling and type I IFN-mediated immune activation (92). In doing so, ADAR1 contributes to the formation of an immune-cold microenvironment, allowing tumor cells to proliferate unchecked.

In contrast to ADAR1, ADAR2 functions as a tumor suppressor in GC through its RNA-editing activity (93). A recent study identified a functionally significant editing event in the PODXL gene, a membrane protein involved in cell adhesion and metastasis (93). ADAR2-mediated editing of PODXL mRNA attenuates its pro-tumorigenic behavior, possibly by altering downstream signaling cascades associated with epithelial-mesenchymal transition (EMT) and invasive growth (93). Reduced expression of ADAR2 in GC is associated with a unique RNA misediting phenotype, characterized by loss of site-specific editing in genes involved in differentiation and apoptosis (93). This editing deficiency correlates with poor prognosis, indicating that restoration of ADAR2 function could offer therapeutic benefits (93). This editing deficiency correlates with poor prognosis, indicating that restoring ADAR2 function could offer therapeutic benefits.

In summary, GC presents a complex dual landscape for ADAR enzymes, wherein ADAR1 acts predominantly as a tumor promoter by silencing innate immune responses, stabilizing oncogenic transcripts, and enabling immune evasion. In contrast, ADAR2 functions as a site-specific tumor suppressor, editing key targets, such as PODXL, to impede invasion and progression. This oncogenic–tumor-suppressive dichotomy underscores the importance of context in ADAR biology and highlights the potential of ADAR-targeted interventions whether through inhibition of ADAR1 or restoration of ADAR2—as promising strategies in precision oncology for GC.

#### 2.1.5 GBM

GBM is the most common and severe aggressive intrinsic brain cancer in adults (149, 150). Even after receiving full surgical resection, along with chemoradiotherapy and adjuvant chemotherapy, GBMs continue to be deadly in every single case (151–153). GBM exhibits extraordinary cellular heterogeneity, including stem-like GBM stem cells (GSCs, also known as brain tumor-initiating cells), contributing to therapy resistance and fast recurrence (154–156). There is evidence that ADARs play a key part in the pathophysiology of GBM (11, 19, 26, 96–98, 157, 158), which has been recently discovered. For instance, Galeano et al. demonstrated that ADAR2 exerts tumor-suppressive effects in GBM through A-to-I editing of CDC14B, a dual-specificity phosphatase that negatively regulates cell cycle progression (97). It has been determined that CDC14B is an essential ADAR2 target gene and that restoring ADAR2 function suppresses the development of GBM by altering the CDC14B/Skp2/p21/p27 cell cycle axis. The editing process that ADAR2 mediates increases the expression of CDC14B, which in turn lowers Skp2 levels and promotes tumor suppression (97). ADAR2-mediated editing stabilizes CDC14B expression, downregulating Skp2, an E3 ubiquitin ligase that normally degrades CDK inhibitors p21 and p27. The upregulation of p21 and p27 leads to G1 cell cycle arrest, thereby inhibiting GBM proliferation (97). This mechanistic cascade (CDC14B/Skp2/p21/p27 axis) illustrates a clear pathway by which ADAR2 editing activity constrains tumor cell growth.

In addition, Tomaselli et al. explored the broader role of ADAR2 in shaping the miRNA landscape in GBM (11). Their work revealed that ADAR2 restores the editing and expression of key miRNAs such as miR-221/222 and miR-21, rebalancing the oncogenic versus tumor-suppressor miRNA ratio (11). This editing restores a physiological miRNome and reduces the expression of oncomiRs, thereby attenuating GBM cell proliferation and migration (19). These findings emphasize the post-transcriptional regulatory capacity of ADAR2 in suppressing malignant phenotypes through miRNA biogenesis. Conversely, ADAR1 has emerged as a key pro-tumorigenic factor in GBM, with functions that extend beyond RNA editing (19). Tassinari et al. revealed that the RNA methyltransferase METTL3 promotes ADAR1 expression through m6A methylation of ADAR1 mRNA, increasing its protein levels without altering editing activity (19). ADAR1, in turn, binds directly to CDK2 mRNA as an RNA-binding protein, enhancing its translation and promoting G1/S phase progression (19). This editing-independent mechanism underscores the critical role of ADAR1 in driving cell cycle advancement and tumor proliferation in GBM.

Zhang et al. investigated the role of ADAR3, a brain-specific member of the ADAR family that lacks catalytic activity in GBM, particularly focusing on its prognostic relevance and regulatory influence on RNA editing dynamics (98). Using information from the Chinese GBM Genome Atlas (CGGA) and three validation cohorts (TCGA, REMBRANDT, and GSE16011), the research investigated the expression of ADAR3 and its connection to the development of GBM, the prognosis, and RNA editing, with a specific focus on GRIA2Q607R. The expression of ADAR3 was shown to have a negative correlation with the development of GBM grade, to be greater in neural subtype and IDH1/2 mutant tumors, and to be linked with better outcomes in patients identified as having lower-grade GBM (LGG) (98). Multivariate analysis established its position as an independent prognostic factor, while bioinformatics analyses associated ADAR3 with processes including cell proliferation, angiogenesis, and adhesion. The results indicate that ADAR3 may function as a prognostic biomarker for LGG, facilitating risk stratification and the development of personalized treatment strategies. Furthermore, focusing on ADAR3 pathways or augmenting their activity could signify a promising therapeutic approach to decelerate GBM progression and enhance patient outcomes.

Jiang et al. further elaborated on the oncogenic role of ADAR1 in GBM stem cells (GSCs)—a subpopulation responsible for GBM propagation and therapeutic resistance (96). Their study demonstrated that ADAR1 activity is upregulated in GSCs, leading to enhanced global RNA editing. Mechanistically, they identified GM2A, a ganglioside metabolism regulator, as a downstream target of ADAR1 editing (96). GM2 gangliosides are classified as glycans. The involvement of various glycan functions in cancer development has been noted (159, 160). Alterations in these GM2 molecular regulators result in inherited metabolic disorders, including the AB variant and Tay-Sachs disease (161). Edited GM2A sustains GSC self-renewal, possibly by modulating lipid-raft–mediated signaling cascades such as PI3K/Akt, essential for stemness and survival (96). Inactivation of ADAR1 or upstream inhibition of JAK/STAT signaling (via TYK2) significantly disrupts GSC maintenance, highlighting a potential vulnerability in GBM biology.

A study conducted by Yang et al. offers a complete pan-cancer analysis of ADAR1, with a particular emphasis on the importance of ADAR1 in terms of prognostic factors in LGG (162). The research shows the differences in ADAR1 mRNA and protein expression across different types of cancer by using bioinformatics techniques. The study also establishes a correlation between the amounts of transcripts and the burden of tumor mutations, immune infiltration, and patient outcomes (162). A three-gene signature (ADAR, HNRNPK, and SMG7) was discovered by Yang et al., responsible for stratifying LGG patients into risk groups. High-risk individuals had a worse chance of survival and higher tumor grades (162). Gene ontology analysis showed that ADAR-related genes are involved in mRNA-binding processes, and upstream regulators, such as SPI1 and miR-206, are associated with increased patient survival (162). The hypermethylation of promoter regions in signature genes and the consistent drug susceptibility patterns underscore their significance in prognosis and therapy. This investigation expands upon previous studies that associate ADAR1 with the advancement of cancer, particularly focusing on its RNA editing functions.

Moreover, Cesarini et al. reported that diminished ADAR2 protein levels correlate with poor prognosis in GBM patients (26). A number of ADAR2 substrates have been recognized as pivotal in GBM, influencing cell cycle checkpoints (11, 97) or altering cell migration and invasion dynamics (163, 164). Functional assays demonstrated that ADAR2 knockdown enhances proliferation, migration, and anchorage-independent growth while also affecting the editing landscape of multiple RNAs involved in cytoskeletal remodeling and mitotic regulation (26). The results underscore the significant prognostic implications of the ADAR2 protein and its involvement in the pathology of GBM. These findings suggest that ADAR2 regulates specific tumor suppressor genes and acts as a global modulator of cellular behavior, the loss of which accelerates GBM progression. This investigation extends previous findings regarding diminished ADAR2 RNA levels in GBM yet distinguishes itself by concentrating on the variability of protein levels across different patients (97, 163, 165, 166).

In summary, ADAR enzymes play context-dependent and mechanistically diverse roles in GBM. ADAR2 functions as a tumor suppressor by editing targets, such as CDC14B, and modulating the miRNA network to restrain cell cycle progression and migration. In contrast, ADAR1 acts as an oncogene, promoting tumor cell proliferation through RNA editing and non-catalytic functions such as CDK2 mRNA stabilization and GSC maintenance via GM2A. These mechanistic insights demonstrate that ADARs are not merely modulators of immune evasion but are intimately involved in regulating tumor cell growth, reinforcing their relevance as therapeutic targets in GBM.

#### 2.1.6 Acute myeloid leukemia

AML represents the most prevalent type of acute leukemia observed in the adult population. It constitutes approximately 1% of all cancers globally and exhibits a greater age-adjusted incidence in developed areas, including Western Europe and Australasia. Over the past several decades, there has been a noted rise in AML, likely attributable to advancements in diagnostic methodologies and the demographic shift toward an older population (167). Recent progress in understanding the molecular mechanisms underlying AML has led to the development of novel therapies specifically targeting the involved genetic mutations and pathways (168). Evidence has demonstrated that ADARs participate in the pathogenesis of AML (33, 99–103). RUNX1 (Runt-related transcription factor 1) is an essential transcription factor that plays a central role in hematopoiesis, significantly impacting the regulation of blood cell differentiation and maturation. The relation to AML is well established since mutations and translocations of RUNX1 are common in many subtypes of this malignancy (169, 170). For example, Guo et al. have illustrated that the RNA-editing enzyme ADAR2 experiences selective downregulation in core-binding factor AML (CBF AML) associated with t(8;21) or inv(16) translocation. This decreased level is caused by the RUNX1-ETO exon 9a fusion protein, which adversely affects the RNA editing activity of ADAR2, a crucial factor in suppressing leukemogenesis. Functional studies showed that ADAR2-regulated targets, including coatomer subunit α, inhibit clonogenic growth in AML cells, pinpointing the vital role of ADAR2 (103). The findings introduce a novel mechanism of ADAR2 dysregulation and its contribution to AML pathogenesis, opening avenues for possible therapeutic interventions.

On the other hand, ADAR1 has emerged as a potent oncogenic driver in AML, acting through multiple complementary mechanisms beyond canonical RNA editing. Balaian et al. showed that ADAR1 is highly expressed in leukemia stem cells (LSCs) and essential for survival (102). Inhibition of ADAR1 using the small molecule Rebecsinib led to a significant reduction in LSC viability and disrupted the leukemia-supportive stromal niche without affecting normal hematopoietic stem and progenitor cells (102). Using a 3D nanobioreactor system, they showed that the ADAR1 inhibitor Rebecsinib effectively reduced LSC survival while sparing normal hematopoietic stem and progenitor cells. Pre-treatment of AML stroma with Rebecsinib disrupted LSC maintenance, reduced key regulatory transcripts, and altered RNA editing, supporting its potential role in reversing malignant niche remodeling (102). This selective dependency of LSCs on ADAR1 activity highlights its role in sustaining leukemic growth and self-renewal.

Mechanistically, Ma et al. revealed that ADAR1 contributes to leukemic persistence by regulating alternative splicing of its own isoforms, particularly favoring the interferon-inducible p150 isoform, which is associated with increased leukemogenic capacity (101). Humanized AML mouse models showed that Rebecsinib significantly prolonged survival compared to vehicle controls and alternative treatments, including Fedratinib (101). The inhibition of ADAR1p150 isoform switching conferred a competitive advantage to normal hematopoietic stem cells over LSCs in the bone marrow niche (101). These findings demonstrated the therapeutic potential of targeting ADAR1 splicing to disrupt LSC persistence and improve AML outcomes. In another notable study, Rodriguez et al. employed genome-wide CRISPR-Cas9 screening and found that TP53-mutant AML cells exhibit a heightened dependency on ADAR1 activity (100). The loss of ADAR1 in these cells led to the accumulation of endogenous dsRNAs, activation of pro-inflammatory signaling pathways, and immune-mediated apoptosis (100). This suggests that ADAR1-mediated editing helps leukemia cells evade innate immune detection, thereby enabling their unchecked proliferation in a high-mutation, high-stress genomic context (100).

Beyond its editing functions, ADAR1 also promotes AML progression through miRNA maturation pathways. Shi et al. demonstrated that ADAR1 interacts with pri-miR-766 and enhances the generation of miR-766-3p, which upregulates WNT5B, a non-canonical Wnt ligand (99). Activation of the Wnt signaling pathway by WNT5B supports leukemic cell self-renewal and survival (99). This interaction is editing-independent and underscores the role of ADAR1 as an RNA-binding regulator of miRNA biogenesis, further amplifying its oncogenic influence (99).

These findings depict a complex and multifaceted role for ADARs in AML. ADAR2 acts as a tumor suppressor, limiting leukemogenesis through precise A-to-I editing of transcripts. In contrast, ADAR1 functions as a central oncogenic node, supporting AML growth and survival by promoting immune evasion, enhancing Wnt signaling via miRNA regulation, sustaining LSCs, and enabling resistance to cell-intrinsic stress. These activities are mediated through catalytic and non-catalytic mechanisms, positioning ADAR1 as an RNA editor and a global modulator of the leukemic transcriptome and microenvironment. Given its centrality in AML biology and the promising results of selective ADAR1 inhibition, further exploration of ADAR1-targeted therapies, especially in TP53-mutant and stem-cell–driven AML, may offer a new frontier in overcoming therapeutic resistance and achieving durable remissions.

#### 2.1.7 Contextual diversity and shared mechanisms of ADAR activity across cancer types

ADAR enzymes exhibit diverse, and sometimes opposing, functional roles across distinct cancer types, governed by both tumor-specific transcriptomic contexts and shared molecular pathways. This contextual plasticity is exemplified by their dual capacity to act as either oncogenic drivers or tumor suppressors depending on cancer type, cellular milieu, and editing targets. In HCC, ADAR1 plays a predominantly oncogenic role by maintaining redox homeostasis through modulation of the Keap1/Nrf2 pathway, enabling tumor cells to survive oxidative stress (86). Conversely, ADAR2 demonstrates tumor-suppressive activity via editing of transcripts like COPA, switching its function from oncogenic to suppressive by inhibiting PI3K/AKT/mTOR signaling (88). However, mutations in ADAR2 may reverse this role and contribute to oncogenesis (87). In CRC, ADAR1 enhances tumor progression through AZIN1 editing, stabilizing c-Myc and promoting IL-8–mediated angiogenesis (91), while also activating FAK/AKT signaling and inhibiting ferroptosis (9). These combined effects highlight the multifaceted carcinogenicity of ADAR1 in colorectal cancer. In GBM, ADAR2 exerts clear tumor suppressor roles through editing targets such as CDC14B and regulating miRNAs, reducing cell proliferation and migration (11, 97).

In contrast, ADAR1 supports GBM stemness and cell cycle progression through editing-independent mechanisms, including direct binding to *CDK2* mRNA and sustaining GM2A signaling (19, 96). In BC, particularly in triple-negative subtypes, ADAR1 acts beyond RNA editing. It stabilizes pro-tumorigenic KYNU protein (95), promotes cancer stemness via WNT/β-catenin signaling (136), inhibits ferroptosis through the miR-335-5p/GPX4 axis (20), and modulates innate immunity via DHX9 interaction (42). In GC, ADAR1 promotes immune evasion through suppression of dsRNA sensing and editing of immunogenic substrates, while ADAR2 suppresses EMT and invasion by editing PODXL (93). These divergent functions underscore the dualistic landscape of ADAR-mediated RNA editing in GC. In AML, ADAR1 supports leukemic stem cell survival and immune escape through editing-dependent and -independent mechanisms, including regulation of Wnt signaling via miR-766 (171) and splicing modulation of its own isoforms (101). In contrast, ADAR2 acts as a tumor suppressor in core-binding factor AML by editing transcripts, such as *COPA*, and restricting clonogenicity (103).

In summary, ADAR1 is recurrently associated with tumor-promoting functions across multiple cancer types—via editing of oncogenes (AZIN1, GM2A), suppression of innate immune signaling (PKR/MDA5), and stabilization of key proteins (KYNU). ADAR2, in contrast, predominantly acts as a tumor suppressor, particularly in neural-origin tumors (GBM) and specific epithelial cancers (HCC, GC, AML), by restoring transcriptomic fidelity and editing tumor suppressor pathways. This context dependency reflects not only differential expression levels and isoform usage but also editing-independent roles that redefine ADARs as multifaceted regulators of tumor progression and immune evasion. Understanding this mechanistic duality is essential for developing isoform- and context-specific ADAR-targeted interventions—whether via inhibition of ADAR1’s pro-tumor functions or restoration of ADAR2’s editing activities.

## 3 The interplay between innate and adaptive immunity in cancer

The immune system has a dual function in cancer, as it not only protects the host against the formation of tumors but also, paradoxically, facilitates the advancement of tumors under specific situations (172–174). The interaction between innate and adaptive immunity mediates this dynamic equilibrium between immunological surveillance and immune escape. Understanding these interactions is crucial for the development of effective immunotherapies. The innate immune system functions as the primary barrier against malignancies. Cells of the innate immune system, including NK cells, DCs, macrophages, and neutrophils, are essential for promptly identifying and removing abnormal cells (175–177). For instance, NK cells discern and eradicate tumor cells deficient in major histocompatibility complex (MHC) class I molecules through activating receptors, including NKG2D (178, 179). Adaptive immunity offers defense against infectious and malignant diseases. The effects are mediated by lymphocytes that detect and respond with targeted precision to disturbances caused by pathogens and tissue damage (180). Lymphocytes, particularly T cells and B cells, are the fundamental cellular components of adaptive immunity (181, 182). Traditional αβ T cell populations are categorized into CD4+ helper T cells and CD8+ cytotoxic T cells. CD4+ T cells perform various effector functions facilitated by both soluble components and cell connections. The primary mechanism by which CD8+ T lymphocytes exert their influence is the destruction of certain target cells. In addition to the production of soluble effector molecules known as antibodies, B cells can also perform the role of antigen-presenting cells (APCs), which are responsible for presenting certain antigens to T cells (180). Cytotoxic T lymphocytes (CTLs) identify tumor antigens displayed on MHC class I molecules and facilitate direct tumor cell destruction through perforin and granzyme (183, 184). IFNγ is produced significantly by macrophages, activated CD8 T cells, natural killer T cells, and Th1 CD4 T cells (185, 186), whereas Tregs may inhibit anti-tumor immunity (187). Macrophages and DCs within the innate immune system are responsible for processing and presenting tumor antigens to T cells, thereby initiating adaptive immune responses. Tumors exhibit a remarkable capacity to circumvent immune system assaults via various mechanisms, such as limiting antigen recognition, suppressing immune responses, and promoting T cell exhaustion. Moreover, tumors possess the ability to obstruct or elude the immune system through the accumulation of specific metabolites and signaling molecules within the TME or by limiting the availability of nutrients to immune cells (7). For instance, these oncogenic pathways or gene mutations upregulate programmed cell death ligand 1 (PD-L1) during cell transformation and tumorigenesis to weaken immune cell activity (7). The TME also further increases the niche for cancer immune escape by boosting the expression of PD-L1 induced by pro-inflammatory cytokines, including IFN-γ, TNF-α, and IL-6 (188–190). A third way is through the induction of immunosuppressive environments, including Tregs and myeloid-derived suppressor cells (MDSCs) (191, 192). The balance and interaction between innate and adaptive immunity are central to the function of the immune system in the context of cancer and its therapies. These dynamics, if understood, can provide a means to overcome immune resistance and achieve durable clinical responses in cancer treatment.

### 3.1 The mechanistic interplay between ADARs and innate immunity in cancer

Innate immunity is the first line of defense in the body against foreign agents such as viruses, and importantly, it plays a vital role in maintaining homeostasis. Whenever any abnormal foreign pathogen is detected or any foreign endogenous nucleic acid is identified, pattern recognition receptors in dendritic cells and other innate immune cells recognize so-called damage-associated molecular patterns (DAMPs) and pathogen-associated molecular patterns (PAMPs), which further lead to a cascade of signaling mechanisms that, in turn, activate various immune genes, including compositions such as inflammatory cytokines and chemokines, one of the key ones being that of IFN-I (193). The induction of IFN creates an inflammatory antiviral state upon activation of interferon-stimulated genes (ISGs) (194, 195). The interferon signal is indispensable to the process of infection clearance, despite unregulated IFN signaling causing pathology. While PAMPs are present across the membrane from outside, abundant endogenous nucleic acids, such as Alu dsRNAs, activate the immune response, resulting in excessive interferon production and damaging effects. The entry of endogenous nucleic acids into this cascade is prevented by A-to-I RNA editing, Alu RNA degradation, and downregulation through RNA-binding proteins (196). Unedited Alu dsRNAs, but not edited Alu dsRNAs, are potent inducers of interferon regulatory factors (IRFs) and nuclear factor kappa B (NF-κB) transcriptional responses, IL-6), IL-8, and ISGs (197). Unedited Alu RNAs can form dsRNAs recognized by dsRNA sensors, RIG-I, MDA5, and TLR3 and stimulate IRF and NF-kB-driven transcriptional responses (197). For example, ADAR1 primarily edited Alu elements in RNA polymerase II (pol II)-transcribed mRNAs, but not putative pol III-transcribed Alus (198). During the IFN response, ADAR1 blocked translational shutdown by inhibiting hyperactivation of PKR, a dsRNA sensor (198). ADAR1 dsRNA binding and catalytic activities were required to fully prevent endogenous RNA from activating PKR (198). ADAR1 knockout neuronal progenitor cells exhibited MDA5 dsRNA sensor-dependent spontaneous interferon production, PKR activation, and cell death. Thus, human ADAR1 regulates the sensing of self-versus non-self RNA, allowing pathogen detection while avoiding autoinflammation (198). Also, inosine incorporation into dsRNA structures by ADARs destabilizes them because I:U base pairs are less stable than A:U pairs (199, 200). This destabilization prevents the dsRNA from being recognized by dsRNA sensors, such as MDA5, which are involved in innate immune responses (201–203). Without sufficient A-to-I editing, these dsRNA structures remain intact and are recognized by cytoplasmic PRRs such as MDA5 and PKR (204). Upon ligand binding, MDA5 and retinoic acid-inducible gene I (RIG-I) stimulate a signaling cascade through mitochondrial antiviral-signaling protein (MAVS) on mitochondria (205). MAVS activation leads to the translocation of the transcription factors interferon regulatory factor 3 (IRF3) and IRF7 and nuclear factor κB (NF-κB) to the nucleus to coordinate the expression of genes encoding IFNs and pro-inflammatory cytokines, resulting in the activation of hundreds of ISGs (205). PKR phosphorylates eIF2α, thereby inhibiting translation and triggering cellular stress responses (206). A-to-I editing by ADAR1 introduces mismatches into the dsRNA duplexes, destabilizing their secondary structure and thereby preventing recognition by these sensors (28, 203). Thus, loss of ADAR1 activity results in excessive IFN production and inflammatory responses due to the accumulation of unedited, immunogenic dsRNAs.

This prevention is essential to avoid an overactive IFN-I response and chronic inflammation, both of which are frequently associated with tumor progression or suppression, contingent on the specific type of cancer. Gannon and colleagues identified ADAR1 as an essential gene for the viability of certain cancer cell lines, as demonstrated through comprehensive genome-scale loss-of-function analyses (207). Cells reliant on ADAR1 exhibited increased expression of interferon-stimulated genes, whereas the absence of ADAR1 led to the activation of the dsRNA sensor PKR, culminating in cell death (207). It has been revealed that the catalytic and non-enzymatic roles of ADAR1 are essential in averting cell death mediated by PKR (207). The findings underscore the potential of ADAR1 as a therapeutic target in certain malignancies. Liu et al. discovered in a separate study that the targeting of the androgen receptor (AR) significantly improves the efficacy of NK cell-mediated cytotoxicity against bladder cancer (BCa) cells by reducing PD-L1 expression (208). This reduction occurs through the decreased levels of circ_0001005, facilitated by the RNA-editing enzyme ADAR2. The AR/ADAR2/circ_0001005 pathway influences PD-L1 through the sequestration of miR-200a-3p, consequently facilitating immune evasion (208). The findings underscore the promise of focusing on the ADAR2/circ_0001005/miR-200a-3p/PD-L1 pathway to enhance antitumor immunity and augment the effectiveness of immunotherapy in BCa.

Recent work by Chan et al. has provided a single-cell resolution map of RNA editing landscapes in lung adenocarcinoma (LUAD), offering novel insights into the heterogeneity of editing patterns and their clinical implications (209). By applying single-cell RNA sequencing (scRNA-seq) to primary LUAD biopsies, the study revealed that therapy-resistant tumor cells exhibited significantly higher levels of RNA editing, particularly at sites enriched in genes associated with drug response and innate immune signaling pathways, such as those involved in interferon responses and antiviral defense (209). Although the study did not perform enzyme-specific profiling, the elevated editing levels observed in resistant subpopulations are most likely mediated by ADAR1, particularly its interferon-inducible p150 isoform, given its dominant role in peripheral tissues and prior reports of ADAR1 upregulation in LUAD (12, 210).

Chan et al. reported a positive correlation between RNA editing burden and somatic mutation load, suggesting a potential link between transcriptomic plasticity and genomic instability (209). Several mechanistic hypotheses may explain this association. First, ADAR1-mediated RNA editing promotes cellular adaptability by generating transcriptomic diversity, which could facilitate the clonal selection of genetically unstable cells under treatment pressure. Second, ADAR1 is known to suppress innate immune sensors such as MDA5 and PKR (211, 212), thereby allowing the immune escape of hypermutated clones that would otherwise be targeted by immune-surveillance. Third, ADAR1 may contribute to genomic instability indirectly by editing transcripts involved in DNA repair and oxidative stress response pathways (e.g., Keap1/Nrf2 axis) (86). By providing single-cell resolution data, this study uniquely demonstrates that RNA-level alterations can precede or accompany DNA-level mutations, influencing tumor evolution dynamically. While the association between high RNA editing load and poor prognosis in LUAD patients marks a significant advancement in the field, the study’s lack of direct ADAR isoform quantification and reliance on correlation-based analyses indicate the need for further mechanistic validation.

Lin et al. studied the function of ADAR1, an RNA-editing enzyme, in macrophages and its impact on tumor development when coupled with IFN-γ (17). Using single-cell RNA sequencing and animal models of lung cancer, melanoma, and colon cancer, the research investigated the effect of conditional ADAR1 deletion in macrophages on the TME (17). At the mechanistic level, the loss of ADAR1 resulted in alterations to the secretion profiles of cytokines. Specifically, it decreased the levels of angiogenic factors (CCL20 and GDF15) and immune-suppressive molecules (IL-18BP and TIM-3) while simultaneously boosting the levels of pro-inflammatory cytokines, such as IFN-γ (17). As a result of these modifications, the cytotoxicity of CD8+ T cells was increased, and angiogenesis was inhibited, which significantly reduced tumor growth in mouse models. The therapeutic potential of combining ADAR1-deficient macrophages with IFN-γ was demonstrated in preclinical models (17). This highlights the function that ADAR1 plays in altering the tumor’s microenvironment to suppress tumor immunity. Consistent with prior research highlighting the role of ADAR1 in cancer immunity, particularly its modulation of interferon signaling in cancer cells (207), the findings of Lin and colleagues align with these observations.

On the other hand, this work broadens the scope to include macrophages, which is a component of ADAR1’s immunological capabilities that is often neglected. The research contributes significantly to a more comprehensive knowledge of immune regulation by establishing that the deletion of ADAR1 in macrophages causes a change in cytokine production favorable to an anticancer milieu. Unlike previous studies that primarily focused on tumor-intrinsic functions of ADAR1, this study highlights the role of macrophage-mediated immunity (209). Moreover, the study relies on animal models and a limited number of cell lines, which limits generalizability to the nature of human tumors and is not broad enough to investigate different interactions within the TME. In summary, there is strong evidence from Lin and colleagues that ADAR1 loss in macrophages, especially combined with IFN-γ treatment, reprograms the TME to inhibit tumor growth. It indicates the therapeutic potential of targeting ADAR1 in innate immune cells and opens the way for new combination therapies in cancer treatment.

In a recent study, Gan et al. explored the role of ADAR1-mediated RNA editing in maintaining immune tolerance within the liver and its implications for tumor immune evasion (30). Normally, ADAR1 prevents the sensing of dsRNA by MDA5, thereby establishing immune tolerance. While the elimination of Ifih1, which encodes MDA5, alleviates embryonic lethality in ADAR1-deficient mice, these mice ultimately face early postnatal mortality. Furthermore, the ablation of additional MDA5 signaling components fails to yield a comparable rescue effect (30). In a liver-specific ADAR1 knockout (KO) murine model, the elimination of MDA5 does not alleviate the hepatic abnormalities resulting from the absence of ADAR1. The resultant Ifih1; ADAR double knockout (dKO) hepatocytes exhibit an accumulation of endogenous double-stranded RNAs, which incites a pronounced inflammatory response and facilitates the recruitment of macrophages to the liver (30). The study elucidates the role of progranulin (PGRN) as a key mediator in the liver pathology resulting from ADAR1 deficiency. It highlights how PGRN enhances interferon signaling and recruits EGFR+ macrophages, intensifying hepatic inflammation (30). It is noteworthy that the PGRN-EGFR axis, along with the related immune responses, experiences considerable suppression in tumors characterized by high levels of ADAR1. This indicates that pre-neoplastic or neoplastic cells may leverage ADAR1-dependent immune tolerance to promote immune evasion (30). The study concludes that the PGRN-EGFR crosstalk is a critical pathway in ADAR1-mediated immune regulation in the liver and highlights its role in tumor immune evasion. The study uncovers a novel PGRN-EGFR signaling axis that operates independently of the well-characterized MDA5 pathway in ADAR1-deficient livers. This expands the current understanding of ADAR1’s role in immune regulation beyond its interaction with MDA5. By linking ADAR1-mediated immune tolerance to tumor immune evasion, the study offers potential therapeutic targets for enhancing cancer immunotherapy. Suppression of the PGRN-EGFR axis in ADAR1-high tumors may be an approach toward overcoming immune evasion mechanisms. The study builds on prior work showing a role for ADAR1 in suppressing aberrant immune activation through MDA5 inhibition (207), but it diverges to show that in the liver, ADAR1 deficiency activates inflammatory pathways independent of MDA5, specifically through the PGRN-EGFR axis. This adds a new dimension to understanding ADAR1’s functions in immune regulation. Furthermore, the relation of ADAR1 to tumor immune evasion via suppression of the PGRN-EGFR axis is complementary to previous studies that have delineated the role of ADAR1 in cancer cell-intrinsic immune modulation (209). A limitation of the current study is its reliance on a liver-specific ADAR1 knockout murine model to make any generalizations to other tissues and systemic immune responses. Translational applicability to human liver physiology is uncertain without further validation in human studies. It focuses on macrophage recruitment and interferon signaling at the expense of broader immune cell interactions and the comprehensive impact of ADAR1 deficiency on the liver immune landscape.

This article by Sun et al. discusses how cancer cells, in this case, pancreatic ductal adenocarcinoma (PDAC), downregulate the immunogenicity of retrotransposon expression to evade immune detection (213). They integrated multi-modal data in PDAC patients and identified specific sequences of Alu repeats that can form ddsRNAs, which activate type-I IFN signaling via RIG-I-like receptors (RLRs) (213). These immunostimulatory Alu-derived dsRNAs were inversely associated with pro-tumorigenic macrophage infiltration in advanced tumors. The study delineates two pathways used by PDAC to mitigate anti-tumorigenic signaling: (1) in mutant TP53 tumors, the LINE-1 ORF1p protein binds and suppresses Alu expression; and (2) in wild-type TP53 tumors, ADAR1-mediated RNA editing reduces dsRNA formation (213). One of the most important roles that LINE-1 ORF1p and ADAR1 play in tumor adaptation to retrotransposon-associated immunological stress is shown by the fact that depleting either of these proteins decreased tumor development *in vitro* (213). This finding is consistent with previous research highlighting the function that ADAR1 plays in lowering the immunogenicity of dsRNA (214, 215) and the contribution that LINE-1 makes to cancer advancement (216, 217). It provides evidence to support the idea that RIG-I activation can induce anti-tumor immune responses. The results highlight the importance of retrotransposon regulation in the TME and immune evasion. The present study provides insight into how tumor heterogeneity may affect immune signaling pathways by showing that PDAC can use different mechanisms depending on the TP53 status. Identifying Alu repeats as a source of dsRNA-mediated immune activation highlights a potential vulnerability in cancer cells that could be therapeutically exploited. Furthermore, the work gives insight into the more general phenomenon of how tumors adapt to the inflammatory stress caused by oncogenic transformation. It suggests that comparable processes may be at play across various forms of cancer. The results of this work provide a complete perspective of how PDAC cells adapt to immunological stress produced by retrotransposons and suggest prospects for new therapeutic strategies.

Nasopharyngeal carcinoma (NPC) is a cancer that most commonly arises in the nasopharynx, which is the upper part of the throat behind the nose (218). This type of cancer is most common in Southeast Asia and has a robust association with infection by the Epstein-Barr virus (219). Xu et al. found that the ADAR1-regulated miR-142-3p/RIG-I axis played a suppressive role in the antitumor immunity of NPC (220). Through utilizing miRNA sequencing, tumor microarrays, and tissue sample analysis, the researchers identified significant upregulation of miR-142-3p in recurrent NPC, which is closely linked to a notably poor prognosis. Functional and mechanistic investigations have elucidated that miR-142-3p directly interacts with CFL2 and WASL, suppressing malignant phenotypes. The overexpression of CFL2 or WASL counteracted the tumor-promoting effects associated with miR-142-3p (220).

Also, miR-142-3p inhibited the RIG-I-mediated immune defense response by obstructing the nuclear translocation of essential immune modulators, including IRF3, IRF7, and p65. ADAR1 was demonstrated to engage with Dicer, thereby promoting the maturation of miR-142-3p in a dose-dependent fashion (220). The study indicates that the ADAR1-miR-142-3p axis is crucial in the progression of NPC and its strategies for immune evasion, thus underscoring miR-142-3p as a potential prognostic biomarker and therapeutic target in NPC (220). Prior research has linked miRNAs to tumor immunity (221–223), and this study extends those findings to NPC. By elucidating the ADAR1-miR-142-3p/RIG-I axis, this study provides critical insights into the molecular mechanisms driving NPC progression and offers promising directions for improving diagnosis and treatment.

## 4 The mechanistic interplay between ADARs and adaptive immunity in cancer

**Figure 3:**
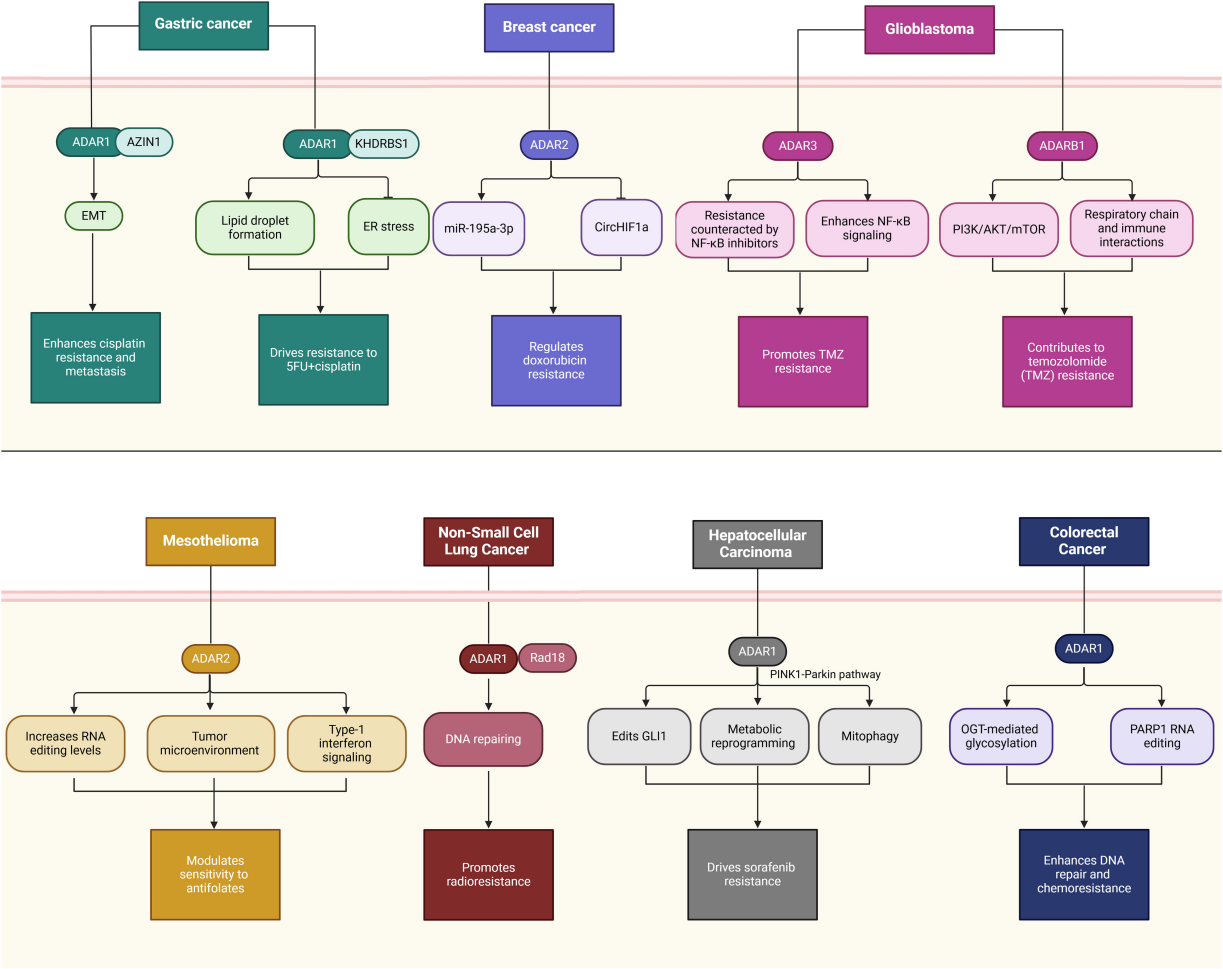
The multifaceted role of ADARs in cancer immunity and immunotherapy. This schematic illustrates the diverse roles of ADAR enzymes—particularly ADAR1 and ADAR2—in modulating immune responses within the TME and shaping outcomes of cancer immunotherapy. For example, ADAR1 is implicated in immune evasion through multiple mechanisms: it intensifies hepatic inflammation via the PGRN-EGFR axis, alters immune cell composition by reducing CD4^+^ T cells and M1 macrophages, and modulates PD-L1 expression to suppress NK cell activity. ADAR1 also suppresses IFN-mediated responses by interacting with PKR and promoting the degradation or editing of immune-related RNAs such as miR142-3p, affecting RIG-I/MAVS signaling and type I IFN responses. Overall, ADARs contribute to immune editing, reprogramming of the TME, and resistance or sensitivity to immunotherapeutic strategies by reshaping innate and adaptive immune responses.

Adaptive immunity is not only necessary to defend the host against infectious and malignant conditions and plays a role in developing autoimmune and inflammatory disorders when pathophysiological circumstances are present (224). In the context of cancer, adaptive immunity is pivotal in inhibiting and facilitating cancer progression (224, 225). The adaptive immune system is fundamental to cancer immunosurveillance since it recognizes and eliminates cells undergoing malignant transformation (226). Principal participants in this process are cytotoxic CD8+ T cells, helper CD4+ T cells, and B cells. CTLs recognize tumor-specific antigens presented on MHC class I molecules and trigger apoptosis in tumor cells (225, 227). Helper CD4+ T cells, by cytokine secretion, facilitate the activation and functionality of cytotoxic T cells and other immune cells, including macrophages and dendritic cells (228, 229). B lymphocytes generate tumor-specific antibodies that aid in macrophage phagocytosis of tumor cells or induce antibody-dependent cellular cytotoxicity (ADCC) (230, 231). ADAR has recently been shown to modulate the interaction between the tumor and the adaptive immune system (**Figure 3**, **Table 2**) (12, 16, 43, 235), which is relevant to the cancer setting. In the case of ovarian cancer, for instance, the research conducted by Gomez and colleagues highlighted a beneficial strategy to reverse immune escape through the combination of DNA methyltransferase inhibitors (DNMTis) with ADAR1 inhibition (236). DNMTis facilitate the transcription of immunogenic double-stranded RNAs, leading to the activation of IFN-I signaling.

**Table 2:** Mechanistic roles of ADARs in cancer immunity and cancer immunotherapy.

Type of immunity	ADAR	Study type	Mechanism	Conclusion	Ref.
Innate immunity	ADAR1	Clinical samples and cell culture	ADAR1-regulated miR-142-3p/RIG-I axis suppresses antitumor immunity	ADAR1-mediated miR-142-3p processing promotes tumor progression and suppresses antitumor immunity.	(220)
Innate immunity	ADAR1	*In vivo*	PGRN-EGFR axis modulates ADAR1-mediated immune tolerance in the liver.	ADAR1-dependent immune tolerance enables immune evasion in liver tumors	(30)
Innate immunity	ADAR1	*In vitro* and *in vivo*	ADAR1 in macrophages inhibits antitumor immunity by regulating cytokine secretion and immune signaling	Loss of ADAR1 in macrophages combined with IFN-γ remodels the tumor microenvironment, enhancing antitumor immunity and suppressing tumor growth.	(17)
Innate immunity	ADAR1	*In vitro*	Prevents PKR activation	ADAR1 prevents PKR activation and suppresses interferon-mediated immune responses in cancer cells.	(207)
Innate immunity	ADAR2	*In vitro*	AR-ADAR2/circ_0001005 axis upregulates PD-L1	Targeting AR-ADAR2/circ_0001005/PD-L1 signaling enhances NK cell-mediated antitumor immunity in bladder cancer.	(208)
Innate immunity	ADARs	The Cancer Genome Atlas (TCGA) database	Elevated ADAR expression modulates immune infiltration by increasing T cell exhaustion and altering dendritic cell and macrophage levels in lung adenocarcinoma.	High ADAR expression is linked to shorter overall survival and poor prognosis in lung adenocarcinoma.	(232)
Adaptive immunity	ADAR1	Patient sample, *in vitro* and *in vivo*	ADAR1 attenuates dsRNA sensing by hyper-editing immunogenic dsRNA, evading MDA5 detection in T-ALL.	Targeting ADAR1 inhibits leukemia-initiating cell self-renewal and prolongs survival in T-ALL.	(43)
Adaptive immunity	ADAR1	Bioinformatics study	ADAR1 in T cells induces immune exhaustion and reduces cytotoxic activity in colorectal cancer.	Targeting ADAR1 in T cells could enhance immunotherapy efficacy in colorectal cancer.	(16)
Adaptive immunity	ADAR1	Patient samples and analysis of data from TCGA	ADAR1 alters immune cell composition, reducing CD4+ T cells and M1 macrophages.	ADAR1 may serve as a promising immune-related molecular target for LUAD patients.	(12)
Immunotherapy	ADAR1	*In vitro* and *in vivo*	ADAR1 suppresses interferon signaling, contributing to immunotherapy resistance.	ATRA-induced ADAR1 degradation synergizes with PD-1 blockade, reprogramming the tumor microenvironment and enhancing antitumor immunity in pancreatic cancer.	(233)
Immunotherapy	ADAR1	*In vitro* and *in vivo*	ADAR1 suppresses innate immune sensing by editing interferon-inducible RNAs, limiting tumor inflammation	Loss of ADAR1 restores immune sensing, overcomes resistance to PD-1 blockade, and enhances antitumor immunity.	(32)
Immunotherapy	ADAR1	*In vitro* and *in vivo*	ADAR1 limits interferon production and contributes to immune checkpoint therapy resistance.	Nanovesicles silencing ADAR1 and blocking PDL1 synergistically enhance antitumor immunity, suppressing tumor growth and metastasis.	(234)

Nevertheless, ADAR1 editing reduces this response (236). The knockdown of ADAR1, in conjunction with DNMTi treatment, markedly improves cytokine production, facilitates CD8+ T cell recruitment, and decreases tumor burden in a mouse model of ovarian cancer (236). The findings indicate a promising approach to alter the immunosuppressive TME and enhance survival rates in ovarian cancer, a condition that generally shows limited response to existing immunotherapies.

A study by Yang et al. examined the role of ADAR1 in lung adenocarcinoma, a common subtype of NSCLC (12). The authors analyzed ADAR1 expression and its prognostic significance utilizing data from The Cancer Genome Atlas and their LUAD cohort. The study employed Kaplan-Meier survival analysis, Cox regression, and multiplex immunohistochemistry to investigate the relationships among ADAR1, LNM, and immune infiltration (12). Findings indicate that ADAR1 is overexpressed in LUAD, correlating with advanced tumor stages, lymph node metastasis (LNM), and a poor prognosis. There was a correlation between high expression of ADAR1 and higher numbers of M0 and M2 macrophages, but a decrease in the number of CD4+ T cells, both indicative of immunological regulation (12). It was shown that the relationship of ADAR1 with CD4+ T cells and M1 macrophages was not positive in the lymph node metastases. Based on these results, ADAR1 can potentially serve as a biomarker of prognosis in LUAD and a conceivable therapeutic target (12). The study aligns with previous research on the prognostic value of immune infiltration in solid tumors (237, 238). It provides new insights into the role of ADAR1 in immune cell dynamics and further details its influence on macrophage polarization and T-cell suppression. However, the findings diverge from previous studies suggesting a broader anti-inflammatory role for ADAR1 (239–241), indicating a complex and context-dependent function. The limitations of the recent study include potential biases from cohort selection, lack of mechanistic insights, and reliance on correlation rather than causation.

Additionally, Hu et al. conducted a study in which they examined the levels of expression of ADARs in LUAD, as well as their prognostic significance and their connection to immune infiltration (232). In this context, Hu and colleagues examined the expression of ADARs in tumor and normal tissues using transcriptome data obtained from TCGA for a cohort of 539 patients with LUAD (232). ADAR overexpression was linked to shorter overall survival, disease-specific survival, and progression-free intervals. Immune infiltration analyses revealed that ADAR expression significantly increased T central memory and helper T cell infiltration while reducing immature dendritic and mast cell infiltration (232). Correlation with markers of T cell exhaustion and tumor-associated macrophages further emphasized ADAR’s role in modulating the tumor immune microenvironment. To provide more explanation, bioinformatics studies were then used to identify the genes and pathways closely related to ADARs, demonstrating the significant role that ADARs play in the development of LUAD (232). The vast datasets used, the strong statistical techniques implemented, and the comprehensive bioinformatics analyses performed, which included pathway enrichment and protein-protein interaction networks, are the strengths of this academic investigation. Limitations of that work include the use of retrospective data, presenting correlational results without mechanistic validation, and reduced translational utility simply due to the lack of empirical models to demonstrate causal relationships.

A study by Zhang et al. investigated the role of ADAR1, an RNA editing enzyme, in CRC, focusing on its expression in immune cells (16). Using RNA editing profiles, bioinformatics analyses, and experimental methods, the researchers demonstrated that T cells, rather than epithelial cells, are the primary source of ADAR1 expression in CRC tissues (16). ADAR1 promotes immune suppression by inducing T cell exhaustion, as evidenced by increased expression of exhaustion markers such as PDCD1, TIGIT, and LAG3. Patients with higher ADAR1 levels in T cells showed poorer responses to immunotherapy, highlighting ADAR1’s role in modulating the TME (16). Based on the observed association between ADAR1 expression and T-cell exhaustion, we hypothesize that ADAR1 may contribute to developing dysfunctional T-cell states through intrinsic and extrinsic mechanisms. Intrinsically, ADAR1-mediated RNA editing might influence the expression and stability of transcripts encoding transcription factors such as TOX, NR4A1, and NFAT, which are known to regulate exhaustion programs in CD8^+^ T cells (242–244). This could affect T cell persistence, effector function, and inhibitory receptor landscape. ADAR1 might shape the immunosuppressive TME extrinsically by modulating IFN-I signaling and cytokine secretion in tumor and myeloid cells, thereby indirectly promoting T-cell exhaustion (32, 207). Moreover, ADAR1 editing activity may affect the expression and presentation of tumor-associated antigens through alterations in MHC-I-related transcripts in tumor cells or dendritic cells (245). Although direct causal evidence remains limited, especially in human T cells, these mechanisms merit further investigation as they may reveal novel targets for reversing T-cell exhaustion and improving immunotherapeutic responses.

T-ALL represents a significant hematological malignancy frequently observed in children, adolescents, and young adults. Approximately 10% to 20% of individuals diagnosed with T-ALL may encounter a relapse several months or even years following the attainment of remission, and they often exhibit resistance to further therapeutic interventions (246, 247). The prognosis for patients with relapsed or refractory conditions is notably unfavorable, with an overall survival rate falling below 25% (248). Patients who have relapsed frequently exhibit increased levels of leukemia-initiating cells (LICs) that possess improved pro-survival and self-renewal abilities. This indicates a potentially susceptible group to applying effective targeted therapies that may have reduced toxicity (249–251). A burgeoning field of inquiry within LIC biology focuses on discovering RNA-modifying enzymes that could collaborate with genetic anomalies to enhance critical LIC functions (252). The recent study by Rivera et al. investigates the role of ADAR1, an RNA-editing enzyme, in T-ALL relapse through its influence on LICs (43). Rivera et al. assessed how ADAR1 facilitates LIC self-renewal by diminishing the detection of immunogenic dsRNA, thereby inhibiting innate immune activation through MDA5. The prominence of A-to-I RNA editing is a defining characteristic of relapsed T-ALL, regardless of the molecular subtype involved (43). The inhibition of ADAR1 markedly diminishes the self-renewal ability of LICs and extends survival in models derived from patient xenografts (43). The study highlights the dependency of LICs on the ADAR1-MDA5 axis and suggests that targeting ADAR1 could be an effective therapeutic strategy to eliminate LICs and prevent relapse in T-ALL. This study aligns with previous research on ADAR1’s role in immune evasion and stemness in various cancers (17, 253). However, it underlines its importance in LIC biology uniquely within T-ALL and extends our knowledge of RNA editing in hematological malignancies. One would expect that the variability in the characteristics of LICs across subtypes of T-ALL might be an important limitation to the generalizability of the findings. It also remains to be explored in long-term studies how targeting ADAR1 could affect normal hematopoietic stem cells and systemic immunity. The design of selective ADAR1 inhibitors and testing of their efficacy in preclinical models of T-ALL should be the focus of future efforts. Research into how ADAR1 interacts with other immune and stemness pathways may identify other therapeutic points of intervention. Expanding studies in patient diversity cohorts and considering combinatorial studies with standard-of-care T-ALL therapies will enhance the clinical relevance of such findings. Practical applications include integrating ADAR1-targeted strategies into relapse prevention protocols and improving outcomes for high-risk T-ALL patients.

Finally, the role of A-to-I RNA co-editing in HCC and its relation to clinical outcomes and immune cell infiltration was investigated by Chen et al. (24). They developed a network focused on RNA co-editing in HCC by applying a multistep algorithm. Their findings indicate that the RNA editing events associated with HCC are predominantly concentrated within this network. Five pairs of risk RNA co-editing events were identified as significantly correlated with overall survival in patients with HCC. Considering the presence of these risk RNA co-editing events, it is possible to categorize patients into high-risk and low-risk groups (24). The high-risk group showed higher levels of exhausted T cells, indicating the differences in immune cell infiltrations between the two groups (24). Besides, seven genes involved in these risk RNA co-editing pairs were found whose expressions effectively distinguish HCC tumor samples from normal tissues (24). These data provide a novel perspective on the etiology of HCC and point toward future therapeutic targets. In line with this, studies presented in existing literature focus on the participation of RNA editing in promoting cancer progression (254, 255). However, the field has advanced by addressing simultaneous RNA editing networks rather than discrete editing events to provide a deeper understanding of the RNA editing landscape in HCC. The added strength of the study lies in the application of state-of-the-art bioinformatics algorithms to construct the co-editing network and integrate the clinical data in validating the findings for their prognostic value. However, several limitations remain, most notably the reliance on computational predictions without experimental validation, which may compromise the accuracy of the identified co-editing events and their biological relevance. It also does not address the functional consequences of identified RNA co-editing events for gene expression and protein function, knowledge that is mechanistically waited for completion. The paper did not include details about sample size and diversity in the patient cohort, so it may not be generalizable to other populations. Therefore, experimental verification of the identified RNA co-editing events and their functional effects on gene expression and protein function in HCC should be the focus of future studies. Further investigation into how these co-editing events shape immune cell infiltration and T-cell exhaustion may help better understand the interaction between tumors and the immune system.

## 5 The mechanistic role of ADARs in cancer immunotherapy

Cancer immunotherapy emphasizes redirecting the focus from tumor cells to the patient’s immune system, facilitating its mobilization and enhancing the activation of the antitumor immune response. This enables immune cells to identify, target, and ultimately eradicate tumor cells (256). Recent indications suggest that ADARs are critical in cancer immunotherapy (32, 208, 233, 234). The research conducted by Ishizuka et al. illustrates that the absence of the RNA-editing enzyme ADAR1 in tumor cells increases their responsiveness to immunotherapy and mitigates resistance to immune checkpoint blockade (32). ADAR1 deficiency reduces the level of A-to-I RNA editing; hence, unedited dsRNA activates PKR and MDA5. These activate inflammation that decelerates tumor growth. This process circumvents the recognition requirement by CD8+ T cells and pinpoints ADAR1 as a critical checkpoint that holds back innate immune responses (32). These findings suggest a novel strategy to overcome resistance to immunotherapies in cancer treatment. In another study, Liu et al. demonstrate that androgen receptor (AR) targeting in bladder cancer enhances NK cell tumor-killing efficacy through reduced PD-L1 expression (208). This is mediated by AR-mediated upregulation of circ_0001005 via the RNA-editing enzyme ADAR2, with circ_0001005 sponging miR-200a-3p to promote PD-L1 expression. Antiandrogen therapy or AR knockdown suppresses this pathway to promote NK cell-mediated tumor clearance (208). These data demonstrate that targeted modulation of the ADAR2/circ_0001005/PD-L1 axis may enhance bladder cancer immunotherapy.

Ding and colleagues present a genetically engineered nanovesicle called siAdar1-LNP@mPD1 in their research. This nanovesicle is intended to improve antitumor immunity by concurrently inhibiting the PD1/PDL1 immune inhibitory axis and silencing ADAR1 (234). The nanovesicle is made up of short interfering RNA against ADAR1 (siAdar1) that is encased in lipid nanoparticles (LNP) and covered with a plasma membrane that is derived from genetically altered cells that have been used to overexpress PD1 (234). This dual-action nanovesicle facilitates ADAR1 silencing in cancer cells, boosting type I/II IFN (IFN-β/γ) responses while presenting PD1 on its membrane to block PDL1 interactions. The result is significant tumor growth regression, prevention of secondary tumors (abscopal effects), and reduced metastasis by globally remodeling the tumor immune microenvironment (234). These findings provide a promising avenue to overcome resistance to immune checkpoint blockade (ICB) therapies. This study defines the role of ADAR1 in immune evasion and resistance to ICBs in consonance with previous research (32). The previous results indicated that silencing of ADAR1 enhances interferon signaling and, thus, tumor sensitivity to immunotherapy (32). Further, PD-1/PDL-1 axis blockade has been the mainstay of effective ICB therapies (257–259). The combination of ADAR1 silencing with PD-1/PDL-1 blockade within a single nanotherapeutic platform represents a novel strategy, overcoming the limitations of single-target approaches with increased therapeutic efficacy. The preclinical models used that cannot accurately represent the heterogeneity seen in human tumors and their microenvironments are a significant limitation of the study. Furthermore, the potential toxicity and off-target effects of siAdar1-LNP@mPD1 have not been explored, raising safety concerns. Lastly, the complexity of nanovesicle engineering may not be scalable and challenging to translate into clinical settings. This study puts forward a revolutionary approach to combat resistance in immunotherapy and opens the way to more effective and personalized treatment options for cancer.

All-trans-retinoic acid (ATRA) demonstrates efficacy in cancer prevention and in treating dermatological conditions and acute promyelocytic leukemia (APL). The pharmacologic impacts of ATRA are primarily facilitated through the engagement of retinoid X receptors (RXRs) and retinoic acid receptors (RARs) (260). Li et al. demonstrated that ATRA could degrade ADAR1 protein and enhance the anti-pancreatic cancer effect of PD-1 blockade (233). ATRA, which was identified in an FDA-approved drug screening, could promote ADAR1 degradation via the ubiquitin-proteasome pathway but simultaneously upregulate PD-L1 expression. When paired with the anti-PD-1 antibody nivolumab, ATRA effectively reconfigured the tumor microenvironment, bolstered antitumor immune responses, and markedly curtailed tumor proliferation in murine models of pancreatic cancer (233). Moreover, a pilot clinical trial revealed that the combination of high-dose ATRA and nivolumab enhanced median overall survival among patients with chemotherapy-resistant pancreatic cancer (233). The study introduces ATRA as the first drug capable of degrading ADAR1, proposing a two-pronged strategy to transform immunologically “cold” tumors into “hot” ones, thereby improving responses to immune checkpoint blockade. The role of ADAR1 in suppressing interferon signaling and promoting resistance to immunotherapy has been reported (32). This study adds to these data, showing a therapeutic benefit by targeting ADAR1. Pancreatic cancer has been notorious for the challenges with immune checkpoint blockade due to the immunosuppressive nature of the microenvironment (261–263). This work aligns with attempts to sensitize “cold” tumors to immunotherapy. The present study diverges from previous approaches in the downregulation of ADAR1 by either RNA interference or genetic editing since ATRA opens a new pharmacological avenue of ADAR1 degradation using an FDA-approved drug with immediate translational potential. The pilot clinical trial of this study had a small sample size, which limited the generalizability of its findings and necessitated the need for larger randomized trials. The safety profile of high-dose ATRA, especially in combination with nivolumab, requires comprehensive investigation to ensure tolerability. Additionally, while ADAR1 degradation and PD-L1 expression were explored, the precise mechanisms of TME reprogramming remain insufficiently detailed, highlighting a need for further mechanistic studies. This study introduces a promising and translatable approach to treating pancreatic cancer by combining ATRA with PD-1 blockade, paving the way for more effective immunotherapies in resistant cancers.

## 6 The mechanistic role of ADARs in drug resistance

**Figure 4:**
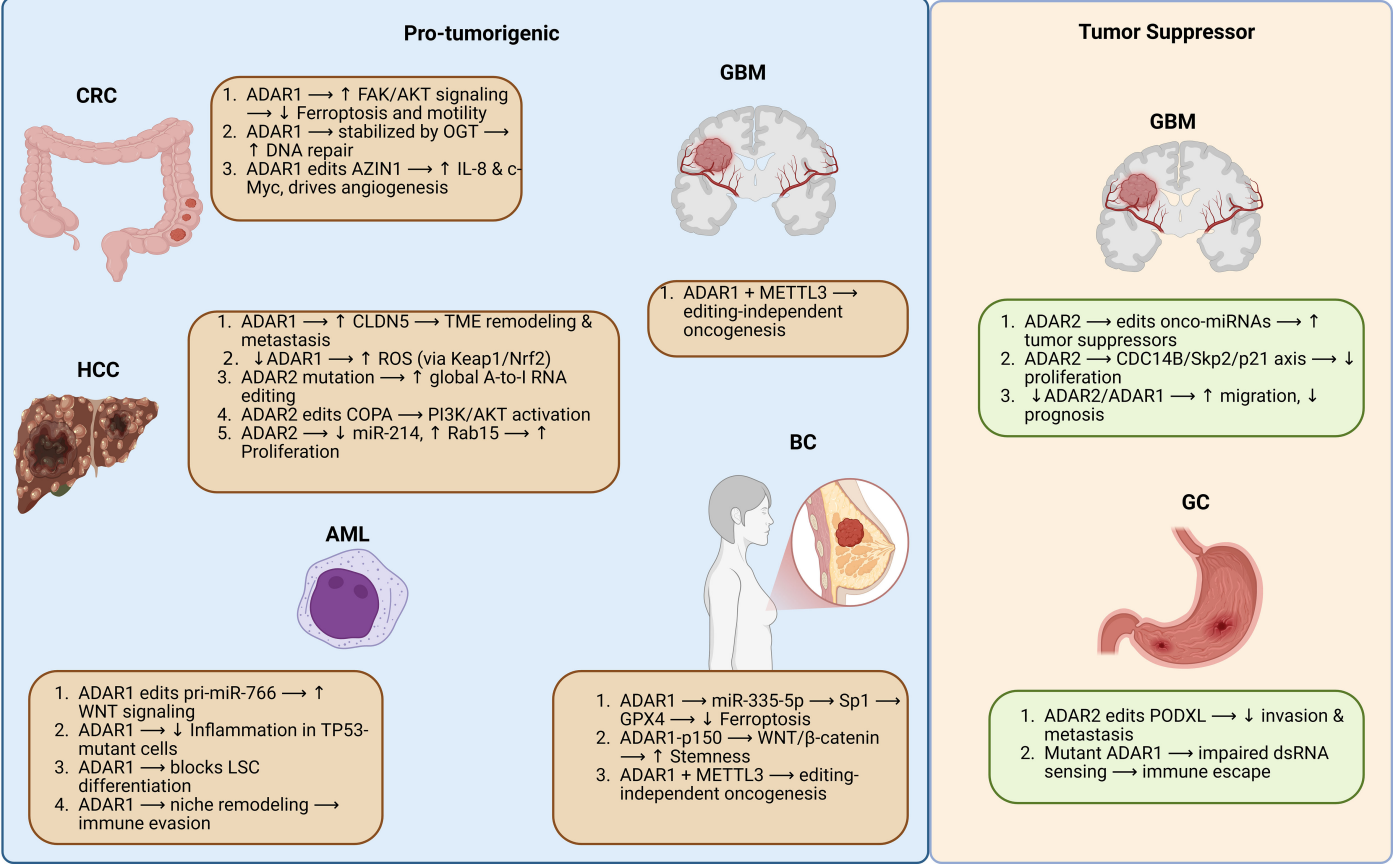
This figure highlights the diverse roles of ADAR family proteins in mediating cancer therapy resistance and tumor progression across various cancer types. For example, ADAR1 promotes cisplatin resistance and metastasis through AZIN1-induced EMT. It also drives resistance to 5-FU and cisplatin via lipid droplet formation and ER stress in coordination with KHDRBS1.

Drug resistance in cancer therapy presents a multifaceted challenge, wherein cancer cells exhibit adaptive mechanisms that enable them to evade destruction by anticancer agents. This complicates the treatment and management of tumor development (264, 265). The emergence of resistance to anticancer therapies significantly elevates the morbidity and mortality associated with malignant tumors (266). Multidrug resistance (MDR) denotes the capacity of cells to develop resistance to an individual pharmacological agent (267). This is a leading cause of chemotherapy failure (268), causing more than 90% of cancer-related fatalities (269). MDR has been associated with various mechanisms (265), including enhanced drug efflux, genetic factors such as gene mutations, amplifications, epigenetic alterations, and microRNA dysregulation (269, 270). Growth factors increased DNA repair capacity and improved xenobiotic metabolism (269). Additional proposed pathways encompass alterations in target molecules (271), dysregulation of cell death systems, intratumor heterogeneity, cancer stem cells, and increased plasticity (270). Recently, it has been demonstrated that ADARs have a significant role in cancer drug resistance (**Figure 4**, **Table 3**) (10, 25, 29, 31, 112, 157). For example, Lu et al. investigated the involvement of ADARB1 in GBM, mainly dealing with chemoresistance to temozolomide (TMZ), a most common problem in GBM therapy (29). Previous studies have shown that the PI3K/AKT/mTOR pathway relates to drug resistance and cancer development in various cancers (274–276). The findings are that ADARB1 is highly expressed in GBM tissues and cells and mediates TMZ resistance via AKT pathway activation (29). Bioinformatics analysis indicated that ADARB1 participated in the mitochondrial respiratory chain and interacted with the tumor–immune system. These results put ADARB1 in place as a prognostic marker and potential therapeutic target for improvement in GBM outcomes.

**Table 3:** Mechanistic roles of ADARs in mediating cancer drug resistance.

ADAR	Associated cancer	Pathway or mechanism	Result in drug resistance	Ref.
ADAR1	Hepatocellular Carcinoma (HCC)	GLI1 editing to enhance mitophagy	Increased sorafenib resistance and reinforced cancer stem cell properties	(272)
ADAR1	Non-Small Cell Lung Cancer (NSCLC)	Interaction with Rad18 and DNA repair pathways	Increased tumor growth and radioresistance	(273)
ADAR1	Gastric Cancer (GC)	RNA editing to stabilize SCD1	Reduced ER stress from chemotherapy and enhanced self-renewal	(10)
ADAR1	GC	Regulation of AZIN1 and EMT	Enhanced metastasis and cisplatin resistance	(31)
ADARB1	Glioblastoma (GBM)	Activation of the PI3K/AKT/mTOR pathway	Increased resistance to temozolomide (TMZ)	(29)
ADAR2	Mesothelioma	RNA editing and regulation of Type-1 Interferon signaling	Enhanced cell proliferation and resistance to antifolate therapies	(112)
ADAR2	Breast Cancer	circRNA-miRNA pathway	Regulation of P-gp expression, reducing drug accumulation and increasing resistance to doxorubicin	(25)
ADAR3	GBM	Activation of the NF-κB pathway	Increased resistance to TMZ	(157)

In another study, Hariharan et al. investigated the role of ADAR2-mediated RNA editing in mesothelioma. They showed that RNA editing is higher in tumors and primary mesothelioma cultures than in normal mesothelial cells (112). The results indicate heterogeneity in RNA editing patterns with higher ADAR2 expression noted in BRCA1-associated protein 1 wild-type tumors. Functional studies have shown that ADAR2 modulates cell proliferation, cell cycle control, and responsiveness to antifolate therapy. It also regulates type-1 IFN signaling and the TME (112). These findings show the dual role of ADAR2 in the growth of mesothelioma and chemoresistance and its modulation of the inflammatory response. A recent study by Kurup et al. investigated the role of ADAR3, an RNA-binding protein with upregulated expression in GBM, compared with normal brain tissue (157). Transcriptome-wide analysis revealed that ADAR3 promotes NF-κB signaling, upregulating 641 genes related to GBM progression. Increased ADAR3 expression was found to enhance glioblastoma cell resistance to temozolomide in an NF-κB-dependent manner, and this effect was reversed by an NF-κB inhibitor (157). These findings underline ADAR3 as a critical driver of GBM growth and chemoresistance by affecting NF-κB signaling.

Cisplatin resistance presents a significant challenge in standard chemotherapy protocols for GC patients and plays a crucial role in influencing their prognosis (277). As a result, there is an urgent need for a dependable biomarker to predict and mitigate cisplatin resistance, explore potential mechanisms of action, and identify possible prevention and treatment targets to improve cisplatin’s efficacy in treating GC (277). The role of ADAR1 in GC metastasis and cisplatin resistance was investigated in a study by Wang et al. (31). Using GC tissue samples and cell lines (AGS and HGC-27, together with their cisplatin-resistant variants), the authors investigated the correlation between ADAR1 and its downstream target, AZIN1, with EMT-related markers by several methods, including immunocytochemistry, western blotting, RNA interference (siRNA), and *in vivo* models of tumor growth (31). Overexpression of ADAR1 and AZIN1 was demonstrated in GC tissues compared to the surrounding non-cancerous tissues (31). ADAR1 knockdown reduced invasion, migration, and proliferation of the cisplatin-resistant cells and downregulated the expression of AZIN1 and EMT markers (31). The combination of ADAR1 with AZIN1 knockdown enhanced these effects, while *in vivo* experiments validated decreased tumor growth and AZIN1 expression (31). This study shows that ADAR1, through AZIN1, promotes metastasis and cisplatin resistance in GC and that its inhibition may be a way to improve the treatment outcome. Wang et al. faced several limitations, such as a small sample size and lack of patient-derived xenograft models, which may limit the generalizability of their findings. Therefore, these findings need confirmation in larger cohorts, and the role of ADAR1 in other chemotherapy-resistant cancers needs further elucidation. Further combination therapy studies on ADAR1 and other EMT-related pathways may fine-tune treatment strategies. Another logical step would be the development of specific inhibitors of ADAR1 and their testing in clinical settings. If successful, such approaches may improve therapeutic outcomes in GC by limiting metastasis and overcoming drug resistance.

The recent investigation conducted by Wong et al. delved into the implications of ADAR1-mediated RNA editing in the context of drug resistance and self-renewal in GC, with particular emphasis on resistance to 5-fluorouracil and cisplatin (5FU+CDDP) (10). Transcriptome research indicated a notable enrichment of Janus kinase/signal transducer and activator of transcription (JAK/STAT) in the resistant line, aligning with research that illustrates the involvement of JAK/STAT in treatment resistance and the characteristics of cancer stemness (278, 279), proposing that transcriptomics or epitranscriptomics aberrations may be more relevant than genomic alterations in conferring chemoresistance. Increasing evidence highlights ADAR1 as a downstream effector of JAK/STAT, enhancing malignant characteristics (96, 280). Wong et al. discovered that JAK/STAT signaling and ADAR1 exhibit upregulation in resistant GC lines using patient-derived organoid models. Whole exome sequencing (WES) and RNA-seq have elucidated that the RNA editing mediated by ADAR1 on stearoyl-CoA desaturase (SCD1) significantly augments its mRNA stability through the enhanced interaction with KHDRBS1. This mechanism stabilizes SCD1, facilitating the formation of lipid droplets to alleviate chemotherapy-induced ER stress while enhancing self-renewal via the upregulation of β-catenin (10). The pharmacological inhibition of SCD1 effectively counteracts chemoresistance and diminishes the frequency of tumor-initiating cells. Clinically, elevated levels of ADAR1 and SCD1 are associated with poorer prognosis, indicating a potential target for addressing GC chemoresistance (10). There are, however, some limitations that have to be addressed. The use of specific organoid lines may limit the generalizability of the findings, as GC is a heterogeneous disease. Although pharmacological inhibition of SCD1 showed promise, potential off-target effects and toxicity of SCD1 inhibitors warrant further exploration. The study did not thoroughly explain the expression of ADAR1; hence, there is still a gap in understanding its upstream drivers. Additional studies are required to address ADAR1 regulation and possible cross-talk with other pathways implicated in GC. Further *in vivo* confirmation of the therapeutic potential of SCD1 targeting awaits models and clinical trials. Combinations of SCD1 inhibitors with standard chemotherapies may offer more effective treatment strategies employing combination regimens.

In a recent investigation, Omata et al. examined the role of the RNA editing enzyme ADAR2 in regulating chemoresistance in murine BC cells via the circRNA-miRNA pathway (25). The researchers created ADAR2-knockdown (Adar2-KD) 4T1 BC cells to investigate how ADAR2 influences susceptibility to the chemotherapeutic agent doxorubicin (25). ADAR2 knockdown led to the upregulation of P-glycoprotein (P-gp), an efflux pump that decreases intracellular drug accumulation, thus promoting chemoresistance. Mechanistically, ADAR2 silencing upregulated circHif1a, which functions as a sponge for miR-195a-3p. The inhibition of miR-195a-3p resulted in the post-transcriptional upregulation of P-gp (25). These findings indicate that ADAR2 inhibits circHif1a biogenesis, allowing miR-195a-3p to inhibit P-gp expression and increase drug sensitivity. The present study aligns with previous findings implicating RNA editing enzymes, particularly ADAR2, in cancer biology and therapy resistance (93, 112). However, it investigates explicitly the circRNA-miRNA pathway to unravel new insights on how RNA editing tunes post-transcriptional gene expression in BC. The use of ADAR2-KD murine BC cells allows the specific role of ADAR2 to be studied in a well-controlled model, and focusing on P-gp brings forth a clinically relevant mechanism of drug resistance. There are, however, several limitations to the study: First, it is based mainly on murine models that may not completely reflect human BC biology. Second, more detailed regulatory mechanisms for ADAR2 expression and its interaction with circHif1a must be elucidated. This would have been taken a step further by validation of the findings using patient-derived tumor samples to make them fully clinically relevant. Future studies should build on this work to include human BC models and determine whether ADAR2’s regulatory role extends to other drug resistance mechanisms or tumor types. Further investigations into possible therapeutic strategies, such as circHif1a inhibitors or miR-195a-3p mimics, might open up new interventions in the fight against.

Although previous studies have already demonstrated the involvement of ADAR1 in tumorigenesis in several cancers (9, 51, 86), the direct association with radioresistance and interaction with DNA repair machinery in NSCLC has remained unclear. More recently, a study by Tian et al. explored the role of ADAR1 in NSCLC, specifically examining its impact on tumor proliferation and sensitivity to radiotherapy (273). They used several approaches, including immunohistochemistry, Western blot, RT-qPCR, RNA sequencing, and numerous *in vitro* and *in vivo* studies, to study the functions of ADAR1 and its molecular interactions. The researchers showed that ADAR1 is overexpressed in NSCLC, which is associated with a worse prognosis in patients (273). Silencing ADAR1 reduced tumor growth and increased radiosensitivity in cell and animal models. Mechanistic analysis demonstrated that ADAR1 interacts with Rad18, a DNA repair regulator, affecting its mRNA expression and localization (273). These data suggest that ADAR1 contributes to tumor growth and radioresistance by interacting with Rad18 and point out the possibility of targeting ADAR1 as a strategy to overcome radioresistance in NSCLC. Several limitations do exist in this study, mainly in sample size; such associations need larger clinical datasets for confirmation. A further potential source of error regards the concentration of Rad18 as a key downstream effector, potentially discounting the influence of other pathways through which ADAR1 affects tumor biology. Finally, there are analysis gaps since the study demonstrates the interaction between ADAR1 and Rad18 but does not explore the overall implications of ADAR1-mediated RNA editing in the TME more extensively. Such addressing could be an agenda for future research to understand the current findings better and enhance our understanding and treatment of NSCLC.

A recent study by Liu et al. investigated how O-GlcNAc transferase (OGT)-mediated glycosylation of the ADAR enzyme drives chemoresistance in CRC (90). Through gain- and loss-of-function experiments, the authors showed that OGT could stabilize ADAR by glycosylation, enhancing its A-to-I RNA editing activity. Using RIP assays, they show that stabilized ADAR enhances the editing of mRNAs involved in DNA damage repair, notably PARP1, thereby enhancing the capacity for DNA repair and resulting in increased resistance to chemotherapy (90). Liu et al. concluded that targeting the OGT–ADAR axis may provide a new strategy to overcome drug resistance in CRC (90). These findings underline the importance of post-translational modifications—in this case, glycosylation in regulating RNA editing pathways- critical for tumor survival under chemotherapeutic pressure. Most studies of OGT have focused on its role in metabolic reprogramming or responses to stress (281, 282); the present study significantly adds a direct mechanism connecting OGT to the regulation of RNA editing. Collectively, this article strongly demonstrates how glycosylation by OGT enhances ADAR-mediated A-to-I RNA editing, increasing DNA repair and promoting chemoresistance in CRC cells. The work points out the need for further research on post-translational modifications that regulate RNA editing to develop new therapeutic strategies to circumvent drug resistance.

Luo et al. explored the role of ADAR1-mediated RNA editing in HCC, especially its effect on liver cancer stem cell generation, maintenance, and aggressiveness (272). GLI1 is a transcriptional effector at the concluding phase of the Hedgehog signaling (Hh) pathway and is meticulously regulated throughout embryonic development and tissue patterning and differentiation (283). Previous studies have also linked GLI1 activity to tumorigenesis and therapy resistance (284–286). The authors identified ADAR1-responsive editing events by RNA sequencing (RNA-seq) and showed that it focuses explicitly on the GLI1 gene. Indeed, the clinical relevance seems to lie in the editing frequency rather than the transcript abundance of GLI1 (272). The specific editing event introducing an arginine-to-glycine substitution at position 701 (R701G) in GLI1 increased the tumor-initiating potential and the self-renewal properties of LCSCs, leading to a more aggressive cancer phenotype (272). Mechanistically, this editing reduced GLI1’s affinity for SUFU, facilitating its nuclear translocation and stabilization by disrupting β-TrCP-mediated degradation.

Furthermore, edited GLI1 induced hyperactivated mitophagy via the PINK1-Parkin pathway, driving a metabolic switch to oxidative phosphorylation, which enhanced stress resilience, stem-like properties, metastatic potential, and resistance to sorafenib (272). These results identify ADAR1 as a vital regulator of LCSC characteristics in HCC through GLI1 editing. The relationship between RNA editing and metabolic reprogramming through mitophagy is relatively new and has been expanding the cancer stem cell biology field. The findings by Luo et al. link molecular events with clinical outcomes such as metastasis and drug resistance, pointing to some potential therapeutic targets. The following are several limitations that somewhat detract from the broader applicability and interpretation of this study: the limited sample size in RNA-seq and clinical correlation analyses may restrict the findings’ external validity and robustness. A second limitation is that, since the focus was mainly on GLI1 as the primary target of ADAR1 editing, other important ADAR1-mediated targets may be overlooked, which could also play a role in liver cancer stem cell behavior. Finally, this study does not provide sufficient information to determine whether GLI1 editing represents a ubiquitous characteristic of all HCC cases or is restricted to certain subtypes, limiting the generalizability of the findings.

## 7 ADARs as prognostic and diagnostic biomarkers

Tumor biomarkers, which are substances produced by tumors or associated with the body’s responses during tumorigenesis and progression, demonstrate significant and promising value in cancer screening and early diagnosis, as well as in predicting prognosis, detecting recurrence, and tracking therapeutic efficacy (287). In recent decades, substantial efforts have been devoted to identifying tumor biomarkers with high sensitivity, specificity, and precision. The latter has massively contributed to the improvement of personalized medicine and the prognosis for cancer patients, largely due to advances in molecular biology technologies in the elucidation of tumor biomarkers (287). Recent studies have shown that ADARs may serve as prognostic markers for cancer patients (12, 93, 125, 288). For instance, Chan et al. examined the role played by ADAR-mediated RNA editing in GC development and outcome (93). In the study, as mentioned earlier, widespread RNA misediting in GC tissues was identified as a consequence of ADAR1/2 dysregulation stemming from genomic alterations. The oncogenic function of ADAR1 and tumor-suppressive functions of ADAR2 were shown, where editing of the PODXL gene by ADAR2 decreased tumorigenicity (93). These findings underline RNA editing as a critical factor in GC and suggest potential therapeutic strategies targeting ADAR enzymes. In another work, Zhang et al. investigated the role of ADAR3 in GBM using data from multiple large-scale datasets (98). They found that the expression of ADAR3 decreased along with the GBM grade and was linked to better prognosis in LGG. Multivariate analysis identified ADAR3 as an independent prognostic factor; further bioinformatics hinted at its involvement in the malignancy of GBM cells through various pathways, including proliferation, angiogenesis, and cell adhesion (98). The study highlighted ADAR3 as a potential tumor suppressor and therapeutic target in GBM.

The study by Yang et al. investigated the role of ADAR1, an A-to-I RNA editing enzyme, in LUAD, a prevalent and deadly form of non-small cell lung cancer (12). Researchers used data from The TCGA and an independent LUAD cohort to analyze ADAR1 expression, its correlation with tumor progression, and its prognostic significance (12). Kaplan-Meier and Cox regression analyses were used to determine the survival outcomes, and multiplex immunohistochemistry was performed to assess the immune cell infiltration in relation to ADAR1 expression. These findings indicated that high ADAR1 expression is related to LNM, late stages of tumors, and poor prognosis (12). In addition, the expression of ADAR1 was correlated with high M0 and M2 macrophages as well as low CD4+ T cells and reduced M1 macrophages, specifically in metastatic lymph nodes (12). These findings position ADAR1 as a prognostic biomarker and potential therapeutic target in LUAD.

A further investigation conducted by Hata et al. identified ADAR1, an enzyme that plays a role in the adenosine-to-inosine RNA editing process, as a predictive biomarker for remnant liver recurrence in patients with liver metastases stemming from CRC who are undergoing hepatic metastasectomy (288). In this group of 83 liver metastatic tissue samples from 36 patients, the expressions of ADAR1 were investigated in comparison with its clinicopathological features and survival outcomes. Elevated expression of ADAR1 was noted in liver metastases from right-sided, synchronous, or RAS-mutant CRC (288). High ADAR1 expression was a strong predictor of remnant liver recurrence, with an AUC of 0.72, and has been proposed as a promising biomarker for identifying patients who may benefit from adjuvant chemotherapy following metastasectomy (288). These efforts may further improve the knowledge and use of ADAR1 as a biomarker to provide better outcomes for CRC patients with liver metastases.

The most recent study by Nakamura and colleagues is the first to investigate the oncogenic role and prognostic significance of ADAR1 in CC (289). The functional role of ADAR1 in three CC cell lines, SiHa (HPV16-positive), HeLa (HPV18-positive), and non-HPV Yumoto, was investigated using ADAR1 knockdown experiments. They have also analyzed the cytoplasmic and nuclear expression of ADAR1 with clinicopathological parameters, including PFS (289). The results showed that ADAR1 silencing was associated with increased apoptosis and necroptosis in all cell lines. Patients with higher expression of ADAR1 in both the cytoplasmic and nuclear compartments had worse PFS, and multivariate analysis confirmed the combination as an independent predictor of prognosis (289). The poorer progression-free survival in patients with high cytoplasmic and nuclear ADAR1 expression may hint that ADAR1 could serve as a biomarker for the aggressiveness of CC and predict poor outcomes. The study concluded that ADAR1 is an oncogenic factor and a potential therapeutic target in HPV-positive and HPV-negative CC.

Qian et al. analyzed the role of ADAR-mediated RNA editing in modulating the poliovirus receptor (PVR) immune checkpoint in CRC (125). Through transcriptome sequencing and experimental verification in two independent Chinese CRC cohorts, increased ADAR and PVR expressions and the positive correlation of the two molecules in CRC tumors were detected9. RNA editing within the PVR 3′-UTR stabilized RNA and hence upregulated PVR expression. Functional studies in HTC116 CRC cells confirmed that modulation of ADAR expression altered PVR RNA editing and expression (125). A diagnostic signature of PVR RNA editing and expression combined showed strong predictive performance in the diagnosis of CRC in both cohorts (125). These findings suggest that ADAR promotes PVR expression and is a potentially novel biomarker in CRC. ADARs have emerged as critical biomarkers in cancer diagnostics and prognostics, opening a perspective on tumorigenesis and therapeutic strategies.

## 8 Therapeutic challenges and considerations in targeting ADARs

While ADARs have emerged as compelling therapeutic targets in cancer, several critical challenges must be addressed before clinical translation. ADAR1 is essential in preventing aberrant activation of innate immunity by editing endogenous dsRNA and suppressing MDA5-mediated interferon responses (28, 202, 203). Complete or non-selective inhibition of ADAR1 can lead to autoinflammatory syndromes (28, 198, 290), as evidenced by embryonic lethality in ADAR1 knockout mice and human Aicardi-Goutières-like phenotypes associated with ADAR1 mutations (291). Therefore, systemic ADAR1 inhibition may cause IFN-driven toxicities in normal tissues, demanding tissue-specific or inducible strategies. Pharmacologic differentiation between ADAR1-p150 and ADAR1-p110, as well as ADAR1 and ADAR2, remains technically challenging due to shared RNA binding and deaminase domains. Isoform-specific splicing or degrader strategies, such as splice-switching oligonucleotides, are being explored to address this issue.

The role of ADARs varies depending on the type of cancer. For example, ADAR1 promotes tumor progression in melanoma and TNBC by evading the immune system and suppressing IFN signaling (292, 293), while ADAR2 exhibits tumor-suppressive roles in GBM and liver cancer by editing transcripts such as COPA and CDC14B (97). This functional dichotomy underscores the need for patient stratification and context-aware interventions. Beyond A-to-I editing, ADARs modulate miRNA processing (11, 85) and bind dsRNAs to sequester them from innate sensors (60, 290). These functions complicate therapeutic inhibition aimed only at catalytic domains, as they may leave non-editing activities intact or disrupt essential protein interactions.

In summary, while ADARs, particularly ADAR1, present exciting opportunities for cancer therapy, the path toward clinical translation remains complex. The essential physiological role of ADAR1 in preventing innate immune activation mandates a highly selective and context-specific therapeutic approach to avoid systemic toxicity. Moving forward, we propose a dual-axis therapeutic model: (1) development of tumor-selective delivery systems, such as ligand-directed nanoparticles or TME-responsive prodrugs, to confine ADAR1 inhibition to malignant tissues; and (2) implementation of isoform-specific RNA-based modulators, such as splice-switching oligonucleotides, to selectively target ADAR1-p150 without affecting p110.

Furthermore, the contextual duality of ADAR functions across cancer types necessitates integrative diagnostic frameworks that combine RNA editing signatures, isoform expression profiles, and immune phenotyping. Such tools will be essential for patient stratification, minimizing risk, and enhancing therapeutic precision. To address the non-catalytic functions of ADARs, especially their roles in RNA scaffolding (76, 294) and miRNA processing (11, 85), we hypothesize that structure-guided allosteric inhibitors or protein-protein interaction disruptors may offer superior specificity compared to conventional catalytic site inhibitors. Finally, we advocate for integrating single-cell RNA editing atlases and machine-learning models to predict editing landscapes and immune phenotypes in individual tumors. This precision oncology paradigm will inform ADAR-targeted strategies and reshape our understanding of RNA editing as a modifiable dimension of cancer immunobiology.

## 9 Conclusion and future perspectives

This review underscores the pivotal roles of ADAR enzymes, particularly ADAR1 and ADAR2, in cancer progression, immune evasion, and therapeutic resistance. Notably, the functions of ADARs are highly context-dependent, acting either as oncogenic drivers or tumor suppressors depending on the tumor type and cellular milieu. The modulation of immune responses, particularly through ADAR-mediated RNA editing of endogenous double-stranded RNAs, is an essential mechanism for tumor immune evasion. Additionally, ADARs’ involvement in modifying drug resistance pathways provides a promising avenue for therapeutic targeting. Future research should prioritize identifying the full spectrum of ADAR1-edited dsRNAs that are critical for MDA5 suppression in specific cancer contexts. By elucidating the particular RNA targets of ADAR1 that regulate immune responses, we can gain deeper insights into how tumor cells evade immune surveillance. These findings could lead to targeted therapies that either inhibit ADAR1 editing or restore immune detection of tumors.

The potential for synthetic lethality through the combination of ADAR inhibitors with DNA-damaging agents warrants in-depth investigation. Given the context-dependent roles of ADARs in cancer—functioning as either oncogenic drivers or tumor suppressors—selective inhibition of ADAR1, in conjunction with conventional chemotherapies or targeted therapies, may enhance therapeutic efficacy. Uncovering how ADAR inhibition can potentiate the effects of existing treatment modalities could yield novel strategies to overcome resistance in refractory cancers. While much of the current focus remains on the RNA-editing activity of ADARs, it is equally essential to delineate their non-catalytic functions, particularly those involved in immune modulation and protein-protein interactions. Further research is needed to elucidate the molecular mechanisms through which ADARs regulate immune cell behavior and contribute to tumor progression independently of RNA editing. A deeper understanding of these non-enzymatic functions may open new therapeutic intervention avenues beyond traditional editing-based paradigms.

The distinct roles of ADAR1 and ADAR2 isoforms across different cancer types remain incompletely understood. Clarifying their specific contributions to tumor progression and immune evasion is essential. In particular, elucidating the opposing effects of ADAR1 and ADAR2 on tumor suppressor pathways and oncogene expression may provide key insights into the molecular pathogenesis of cancer. Given the pivotal role of the TME in modulating immune responses and mediating therapeutic resistance, future research should investigate how ADAR-mediated RNA editing shapes and sustains an immunosuppressive TME. Exploring ADAR interactions with stromal, immune, and endothelial cells could uncover novel targets to enhance the efficacy of immunotherapy. Advancing these lines of inquiry will deepen our understanding of the multifaceted functions of ADARs in cancer biology and support the development of ADAR-based therapeutic strategies aimed at overcoming immune escape and drug resistance.
